# The impact of COVID-19 lockdown on air pollution in Europe and North America: a systematic review

**DOI:** 10.1093/eurpub/ckac118

**Published:** 2022-09-08

**Authors:** Maria Bakola, Ireri Hernandez Carballo, Eleni Jelastopulu, David Stuckler

**Affiliations:** Research Unit for General Medicine and Primary Health Care, Faculty of Medicine, School of Health Science, University of Ioannina, Ioannina, Greece; Department of Public Health, Medical School, University of Patras, Patras, Greece; Department of Social and Political Sciences, Bocconi University, Milan, Italy; RFF-CMCC European Institute of Economics and the Environment, Centro Euro-Mediterraneo sui Cambiamenti Climatici, Milan, Italy; Department of Public Health, Medical School, University of Patras, Patras, Greece; Department of Social and Political Sciences, Bocconi University, Milan, Italy; Department of Social & Political Sciences and Dondena Research Centre, University of Bocconi, Milan, Italy

## Abstract

**Background:**

Multiple studies report reductions in air pollution associated with COVID-19 lockdowns.

**Methods:**

We performed a systematic review of the changes observed in hazardous air pollutants known or suspected to be harmful to health, including nitrogen dioxide (NO_2_), nitrogen oxides (NO_x_), carbon monoxide (CO), sulfur dioxide (SO_2_), ozone (O_3_) and particulate matter (PM). We searched PubMed and Web of Science for studies reporting the associations of lockdowns with air pollutant changes during the COVID-19 pandemic in Europe and North America.

**Results:**

One hundred nine studies were identified and analyzed. Several pollutants exhibited marked and sustained reductions. The strongest was NO_2_ (93% of 89 estimated changes were reductions) followed by CO (88% of 33 estimated pollutant changes). All NO_x_ and benzene studies reported significant reductions although these were based on fewer than 10 estimates. About three-quarters of PM_2.5_ and PM_10_ estimates showed reductions and few studies reported increases when domestic fuel use rose during COVID-19 lockdowns. In contrast, O_3_ levels rose as NO_x_ levels fell. SO_2_ and ammonia (NH_3_) had mixed results. In general, greater reductions appeared when lockdowns were more severe, as well as where baseline pollutant levels were higher, such as at low-elevation and in densely populated areas. Substantial and robust reductions in NO_2_, NO, CO, CO_2_, PM_2.5_, PM_10_, benzene and air quality index pollution occurred in association with COVID-19 lockdowns. O_3_ levels tended to increase, while SO_2_ and NH_3_ had mixed patterns.

**Conclusions:**

Our study shows the profound impact of human activity levels on air pollution and its potential avoidability.

## Introduction

COVID-19 led to rapid and profound changes in modern human activity on a scale never seen in advanced industrialized countries outside of World War II. While some changes directly arose from the pandemic itself and associated fears, the most significant changes came from government responses to COVID-19, especially from implementing so-called ‘lockdown’ measures.^1^ Virtually all European and North American countries, albeit to varying degrees, introduced severe restrictions to human mobility. This included halting international travel, road transit and shutdowns of industrial activity. In the most extreme cases, such as in Italy, people were prevented from leaving their homes altogether except under emergency circumstances. These substantial alterations to human activity have had an impact on air pollution. It is well established that the vast majority of air pollutants are emitted through anthropogenic sources from the use of industry, power stations, combustion engines and vehicle exhausts.^2^ These include particulate matter (PM), black carbon (BC), nitrogen oxides (NO_x_), ammonia (NH_3_), carbon monoxide (CO), methane (CH_4_), non-methane volatile organic compounds, including benzene, certain metals and polycyclic hydrocarbons. Thus, COVID-19 lockdowns create a unique and tremendous opportunity to learn about the potential avoidable effects of human activity on pollution levels. Yet so far, the early reports have been mixed. Several studies suggest that certain pollutants rose during COVID-19, including ozone (O_3_) and NH_3_ levels,^3^^,^^4^ while many suggest there have been substantial declines in PM (albeit with some exceptions)^5^ and corresponding reductions in air quality. However, these studies vary considerably in how they measure air pollution, which pollutants were studied, and whether they attempt to quantify the impact of lockdowns and not just the COVID-19 epidemic. Moreover, reductions in emissions can have markedly varying effects on pollution because of potentially modifying factors, such as emission heights, sunlight, meteorology and geography.^6^^,^^7^

Here, we integrate these disparate studies to provide a comprehensive picture of the effects of COVID-19 lockdowns on air pollution. To do so, we performed a systematic review of studies investigating the impact of lockdowns on the Environmental Protection Agency (EPA) ‘Criteria Air Pollutants’, known to have adverse health effects (available in Supplementary appendix S1), including CO, lead, ground-level O_3_, nitrogen dioxide (NO_2_), PM and sulfur dioxide (SO_2_).^8^ We compared studies across Europe and North America, in view of differing regulatory approaches to pollution. Additionally, we evaluated potential mechanisms and modifying factors for the relationship between lockdown measures and air pollution. Specifically, we aimed to investigate: what are the effects of lockdowns/restrictive measures on air pollution levels during the pandemic in Europe and North America; why did some nations and cities experience greater reductions in air pollution levels; and what gaps remain and merit further investigation.

Our findings are particularly relevant to ongoing discussions about how best to reduce air pollution, as it is a major avoidable risk factor for premature death and currently over 90% of the world’s population lives in areas where air pollution levels exceed the safe thresholds set out in the World Health Organization’s Air Quality Guidelines.^9^

## Methods

### Search strategy

**Figure 1:**
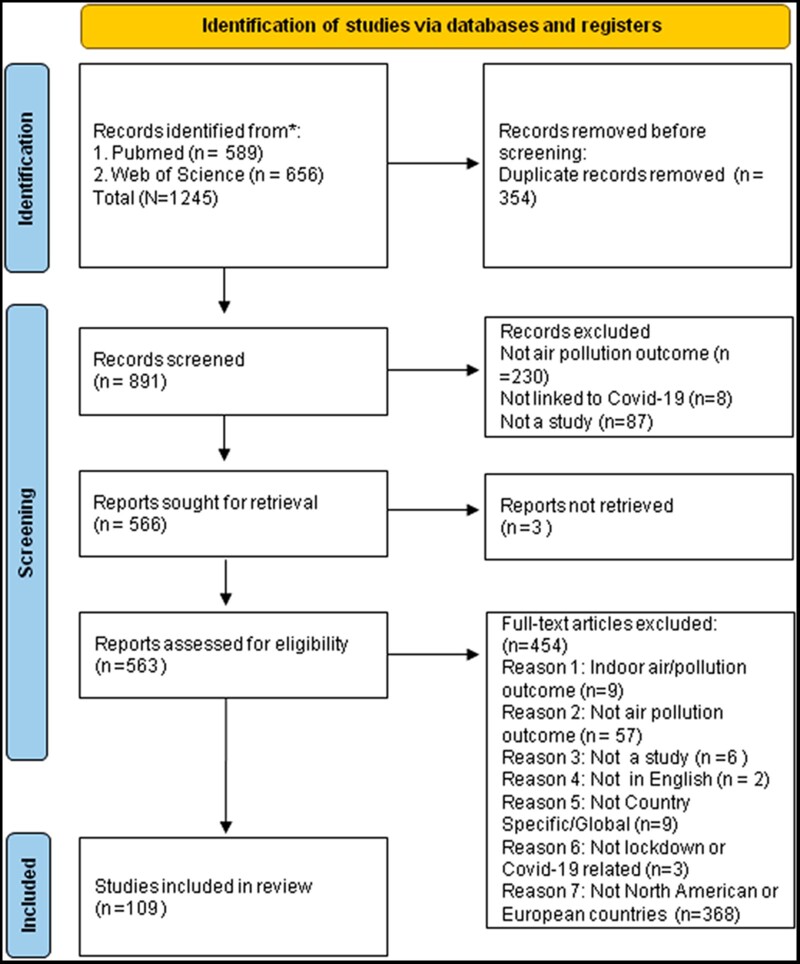
PRISMA study inclusion diagram *Note*: From Page *et al*.^10^

We searched PubMed and Web of Science covering 2020, the onset of COVID-19, through to the time of search, 7 June 2021. A series of keywords were used to capture the three components of the search: ‘lockdown’, ‘COVID-19’ and ‘air pollution’. The full replicable search is detailed in Supplementary appendix S2 and all steps followed the PRISMA (Preferred Reporting Items for Systematic Reviews and Meta-Analyses)^10^ best-practice guidelines for systematic reviews (figure 1).

This initial search resulted in 1,245 articles, of which 589 were from PubMed and 656 from Web of Science. After removing duplicates (*n* = 354), a total of 891 articles remained for screening and eligibility.

### Inclusion/exclusion criteria

We applied a series of inclusion/exclusion criteria, as follows. Articles were included if they: (i) used an observational study design; (ii) included air pollution as an outcome measure; (iii) covered the COVID-19 period; and (iv) included an analysis of the role of lockdown and/or mobility restrictions. We removed articles which were not written in English and did not include data covering pollution in Europe or North America.

Screening of the title and abstracts resulted in the removal of 325 papers, leaving 566 articles. All were successfully retrieved. Further applying eligibility to the full text left a final sample of 109 studies for inclusion into the systematic review study.

### Extraction and analysis

From the 109 articles, we extracted the main data on the study population, time period, design, adjustments for potential confounding factors, baseline for comparison, outcome measures employed and the overall findings and conclusions. Quality assessment was based on two main primary dimensions relevant to observational studies (as taken from the STROBE checklist for observational studies), namely corrections for potential confounding factors and risk of bias.

We investigated potential patterns in findings by disaggregating the studies by geography (Europe and North America) and each EPA pollutant. Then, we sought to identify potential mechanisms of changing pollution levels associated with lockdown (including pollution transport, vehicle emissions and power generation) as well as potential modifying factors based on geography (such as elevation and urbanization) and industrial factors.

Data extraction was conducted independently by two authors and discrepancies were resolved through consensus. This review was registered with the International Prospective Register of Systematic Reviews (PROSPERO CRD42021271914).

## Results

**Figure 2:**
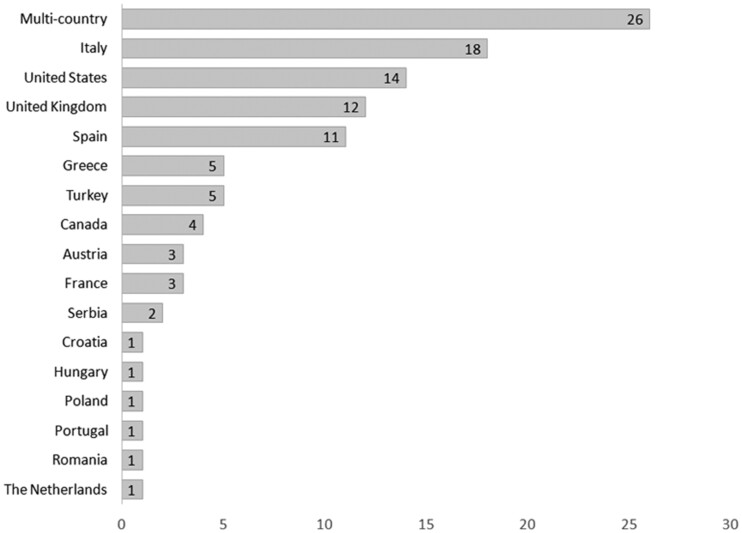
Number of studies by country

Overall, 109 studies were included, estimating a total of 381 changes in pollutants in association with COVID-19 lockdowns. Figure 2 shows the geographic distribution of studies in the analytical sample. There were 18 North American studies, including from the USA (*n* = 14) and Canada (*n* = 4); 67 from Europe, of which Italy and Spain were the most extensively studied countries (*n* = 18 and *n* = 11, respectively); and 26 from multi-country studies including one or a combination of these regions.

All studies included in the review used pre-/post-comparison designs. However, the reference period/baseline varied considerably as did the lockdown period for comparison. Most US studies coded lockdown as 15 March to 30 April 2020, the most stringent period. In Europe, most countries introduced lockdowns in mid-March. However, the lifting of restrictions and their ongoing severity differed substantially across countries and over time.

First, we evaluate the main findings and patterns by pollutant, starting with those which indicated a clear reduction, followed by those with no evident pattern or mixed effect and concluding with those exhibiting adverse/negative effects.

### Air pollutants which were reduced in association with COVID-19 lockdowns: NO_2_, NO, PM_2.5_, PM_10_, CO, CO_2_, benzene, BC and AQI

**Figure 3:**
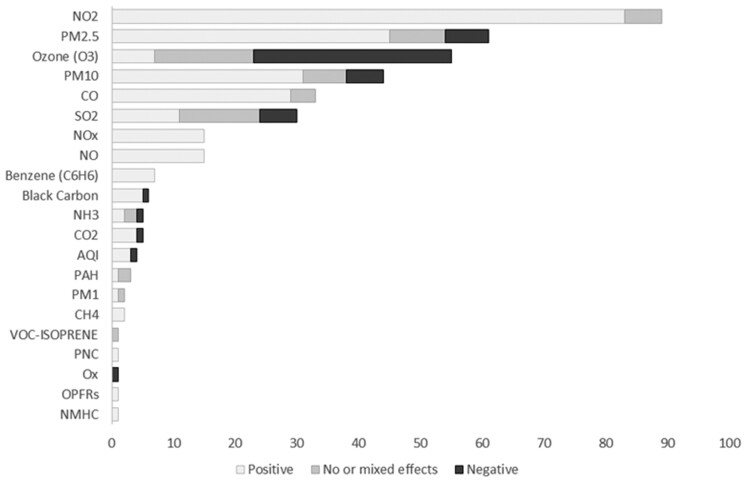
Summary of study findings, 109 studies and 381 estimated pollutant changes, negative = worsening (increasing levels), positive = improvement (reduction in levels), no or mixed effects = mixed/no effect *Note*: AQI, air quality index; PAH, polycyclic aromatic hydrocarbons; VOCs, volatile organic compounds; PNC, particle number concentration; OPFRs, organophosphate flame retardants; NMHC, non-methane hydrocarbons.

Several air pollutants exhibited significant and robust reductions in association with COVID-19 lockdowns, as shown in figure 3. These included NO_2_, NO, PM_2.5_, PM_10_, CO, carbon dioxide (CO_2_), Benzene, BC and air quality index (AQI). CH_4_, particle number concentrations, organophosphates and non-methane hydrocarbons showed reductions, but these were only reported in two studies or less.

Of these, the air pollutant with the strongest and most robust pattern of decline was NO_2_ (83 out of 89 estimates refer to reductions). Among a total of 93 estimates, 10 in North America showed significant reductions and 4 reported no or mixed effects. In Europe, however, all 52 reported estimates were positive, and none was mixed or negative. The reported changes were substantial. For example, one analysis investigating 47 major cities of Spain found NO_2_ dropped by 51% and 36.4% in the lockdown (57 days) and deconfinement (42 days) periods, respectively.^11^ Finally, in the mixed country studies, all but two studies were positive. These declines appeared to be prominently linked to reductions in non-essential vehicles and combustion activities in industrial and commercial sites.^12^

NO, NO_x_ and benzene had universally positive findings. Benzene and NO were only studied in Europe, whereas there were two NO_x_ studies in the USA. These were strong and robust findings; however, the number of studies was smaller than for NO_2_, PM_2.5_ and PM_10_. In Europe, one study in Rome, Italy, showed that NO declines ranged from −34% to −76% in urban traffic sites, due to the largest reductions in road transport and non-essential road activities.^12^ In France, benzene and NO_x_ decreased by 9.28% and 44.5%, respectively,^13^ but these declines were not sustained when lockdowns ended. In the UK, large reductions in NO_x_ were observed up to 47%, which were attributable primarily to reduced electricity demands.^14^ Similar magnitudes of declines in NO_x_ were observed in the USA.^15^

The second-most consistent and strong pattern was found for CO (88% of 33 included estimates). All North American studies reported significant and substantial reductions corresponding to the COVID-19 lockdown periods. At the low end of the range, one analysis of CO levels in Southern Ontario reported ∼20% reductions,^16^ in both major urban and rural areas. One study reported especially large reductions in the Bronx and New York County up to 74.2%.^17^ In Europe, the studies which investigated 18 changes in CO revealed that 15 changes had significant reductions and three changes had mixed or no effect. The studies which involved multiple countries showed seven positive changes and one with no or mixed effect.

PM_2.5_ and PM_10_ had strong reductions; approximately three-quarters of estimated changes (76 out of 105) reported significant declines. As one example, Chadwick *et al*.^18^ found major reductions of up to 71% in the period 11 March to 10 April 2020, which were periods of intense quarantine. Greater reductions were seen in lower elevation and more urbanized areas. These findings were corroborated in Europe. One study from Altuwayjiri *et al*.^19^ in Italy found a 78% decrease in PM_2.5_ during the lockdown phase in comparison to the same period in 2019, mainly due to traffic restrictions. However, several studies reported no significant change in the PM_10_ concentration during lockdown. For example, Varotsos *et al*.^20^ measured PM_2.5_ and PM_10_ at monitoring stations in Athens, Greece, where no significant fluctuations were reported. This may have resulted from compensating meteorological conditions during lockdowns. Finally, one study in Austria found that PM_10_ rose in residential areas. The authors suggested that this occurred as people spent more time at home and used more domestic fuel, as the hours of increased emissions were from 10am to 8 pm. They also found a non-consistent relationship between traffic and PM_10_, which they argued reflected activities to lower traffic intensity prior to COVID-19 lockdowns.^21^

### Air pollutants with null or worsened associations with COVID-19 lockdowns: O_3_, SO_2_ and NH_3_

Of all pollutants under study, O_3_ shows the highest propensity to worsen during lockdowns (32 out of 55 estimates found increased levels). A few studies also found increases in SO_2_ (6 out of 30) and NH_3_ (1 out of 5), but the majority found no effect.

Most of O_3_ studies were conducted in Europe. Collivignarelli *et al*.,^22^ e.g. found sustained large NO reductions but increases in O_3_ levels in Milan. Similarly in the UK, O_3_ concentrations increased by 7.6%, with the largest increases taking place at roadside sites in association with reduced NO emissions.^23^

Turning to SO_2_, several studies note that changes were strongly linked to industrial activity. One study in Florida, USA, found SO_2_ levels rose due to increased power generation during lockdowns. The authors argued this highlighted the need for more sustainable energy sources for power.^24^ As another example, Filonchyk *et al*.,^25^ reported increased SO_2_ levels in Poland due to active burning of fuel, primarily coal, which accounts for all the national SO_2_ emissions. These emissions were also linked to manufacturing facilities, which continued to operate even during quarantine periods. Similarly, Celik and Gul^26^ find in Istanbul that about half of monitoring stations recorded declines, while the other half reported increases. The rises appear correlated with the areas where industrial plants and heavy traffic were located, which in Istanbul make up the primary sources of SO_2_.

NH_3_ was studied only in Europe with mixed results (*n* = 5). One study reported no changes during COVID-19 lockdowns in Italy because NH_3_ emissions were almost entirely driven by emissions from the agricultural and livestock sectors and these sectors were unaffected by the restrictive measures.^27^ Another study in Italy, however, did report an increase in NH_3_ emissions as a result of an increase in agricultural activities of 61.8% in Bologna, 71.2% in Rome and 93.7% in Milan compared to those of the year 2019.^28^ Summary of study findings by geographic region and summary tables of the included studies are available in Supplementary appendices S3 and S4, respectively.

### Mechanisms and modifying factors

**Figure 4:**
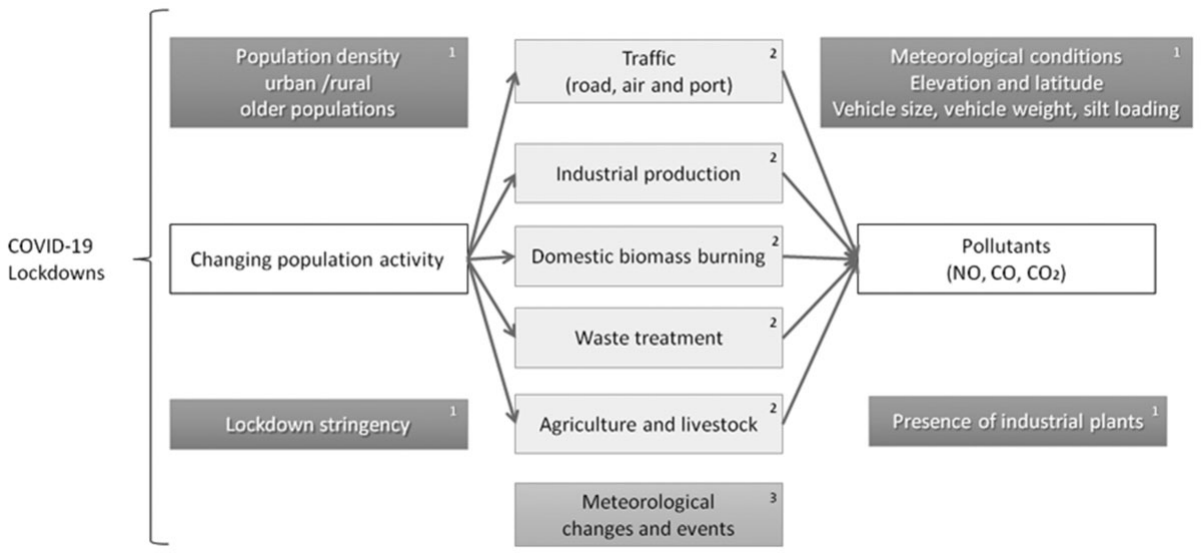
Conceptual framework for mechanisms and modifying factors *Note*: 1 = Potential moderating factors, 2 = Potential mediating factors, 3 = Potential confounding factors.

Several mechanisms were proposed to account for the marked reductions in pollutant levels, as summarized in the conceptual framework of figure 4. These variously included: reductions in transportation, including motor vehicles, locomotives and airplanes; reductions in industrial activity including coal-based power plants; greater sunlight and warmer temperatures; adverse meteorological events, such as sandstorms and wind changes; livestock and agriculture; and seasonal forest fires or wildfires emitting PM.

Few studies performed formal mediation analyses to test the contributions of alternative potential mechanisms. However, important patterns emerged by pollutant. In particular, heavy traffic use was linked to changing patterns of CO, CO_2_, volatile organic compounds (VOCs), NO_x_ and PM pollutants.

- Lockdown reductions in heavy traffic: reduced vehicular emissions were attributable for significant reductions in CO, CO_2_, VOCs, NO_x_ and PM pollutants.

Tian *et al*.,^29^ e.g., report that the estimated CO_2_ from motor gasoline consumption in April 2019 was almost twice than that in April 2020. Several strong studies found that NO_2_, NO_x_ and NO reduced in association with traffic reductions, but resurged as lockdowns were lifted and vehicles returned to roads.^30^

- Lockdown reductions in industrial emissions: as factories and industrial plants were shut down in lockdowns, their emissions declined. These were especially strongly linked to PM, O_3_, NO_2_ and NO pollutants.

Combustion emissions from sources like power generation or road traffic contribute to harmful air pollutants, such as O_3_ and PM_2.5_. Aydin *et al*.^31^ reported higher levels of PM in 2019, due to more than 81 active industrial plants including mining, textile, agriculture and chemical plants at the Erzurum province in Turkey, which were reduced during lockdown. At the same time there was an increase in O_3_ because of PM reduction. In Italy, Bassani *et al*.^12^ found reduction of NO_2_ and NO pollutants due to the reduction of non-essential vehicles and combustion activities in industrial and commercial sites. Lockdown reductions in agriculture and livestock were strongly linked to NH_3_ because NH_3_ can combine with NO_x_ and SO_2_ in the air and form PM_2.5_ and PM_10_.^27^ Ninety-six percent of NH_3_ emissions originate from agricultural activities and form secondary PM_2.5_.^32^

- Lockdown reductions in farming and agriculture.

Querol *et al*.^33^ in their study in Spain report high NH_3_ emissions from farming and agriculture even during the lockdown. Viatte *et al*.^4^ explain that NH_3_ is a precursor of ammonium sulfate [(NH_4_)_2_SO_4_] and ammonium nitrate (NH_4_NO_3_) aerosols, which are formed when atmospheric NH_3_ reacts with sulfuric acid (H_2_SO_4_) and nitric acid (HNO_3_), which is formed in urban areas from the oxidation of NO_x_, mostly emitted by traffic.

- Some rises in secondary pollutants like O_3_, which have inverse relationships to lower emissions of a primary pollutant.

Declining NO levels appeared to account for increases in O_3_, due to the suppression of scavenging of O_3_ through NO_x_ titration.^34^ Fenech *et al*.^35^ showed that during lockdown, the NO_2_ concentrations decrease and O_3_ concentrations increase. This is due to the reduced titration effect of O_3_ by NO, which results in higher O_3_ concentrations.

Finally, several modifying factors were invoked to explain why patterns varied considerably across countries and within them in different cities. The most common factor invoked was the stringency of lockdown measures^36–38^ as well as population density^39^; both were correlated with greater reductions in pollutant levels. Several authors speculated that high elevation areas and less residential areas/rural populations might have smaller absolute decreases, because they started from low baseline pollutant levels.^23^

Other potential modifying factors speculated to account for geographic heterogeneity in pollutant changes included, among others: baseline traffic volume; baseline industrial emissions; uses and mode of domestic energy; and meteorological determinants, particularly wind patterns.

### Quality assessment

Several studies adjusted for potential confounding from weather and atmospheric changes. Most studies attempted to correct for meteorological patterns. These included, wind speed and direction, rain, dry and sunny weather, solar radiation, sunlight hours, atmospheric pressure, temperature, relative humidity, desert dust episodes and other extreme events. Additionally, studies sought to adjust for seasonal variations, either by comparing with historical matched months or by de-trending the data for seasonal time-trends.

As studies were particularly interested in highlighting the role of COVID-19 related changes, many included statistical modeling adjustments for seasonality, wind speed, precipitation, humidity and other meteorological conditions, which were in theory not directly affected by lockdowns. These studies tended to find that, even after adjusting for these factors, there were marked declines in pollutants associated with lockdown periods. Taking NO_2_ as an example, one analysis of 20 cities in North America reported that meteorological conditions were quite favorable so as to lead to independent reductions over the lockdown period. However, even after substantial adjustments for seasonality, wind and other meteorological effects, the authors found that NO_2_ levels dropped significantly between 9% and 43% across US cities in association with COVID-19 lockdown.^40^ Another study in Southern Ontario estimated that only about 25% of changes in NO_2_ could be attributed to seasonal or meteorological changes.^41^

## Discussion

Our review finds that restrictive measures due to COVID-19 pandemic had a mixed effect on air pollution, which is a complex mixture of natural and anthropogenic sources. The pollutants, which showed clear and strong reductions in Europe and North America in association with lockdowns were NO_2_, NO, CO, CO_2_, PM_2.5_ and PM_10_, benzene and AQI. In contrast, O_3_ exhibited an inverse relationship with NO_x_, so that as NO_x_ levels declined, O_3_ levels rose during COVID-19 lockdowns. Finally, there were mixed effects for SO_2_ and NH_3_.

Our systematic review had several important limitations. First, our review was restricted to English language publications. This could potentially miss important country-specific literature on air pollution. Additionally, we did not include national air pollution policy reports from ‘grey literature’, as these too were often in different languages, especially for Europe. However, we were able to capture major published documents from the Centers for Disease Control and Prevention (CDC) and European CDC in our search. Several limitations also arise from the studies included in the reviews themselves. One is that the timing of lockdowns differed considerably across Europe and North America, making it difficult to draw clear cross-national comparisons. However, the included studies sought to adjust for seasonality so as to facilitate comparability. Second, many studies employed varying reference periods, with baselines ranging from the corresponding months of lockdown in preceding years through to historical long-run average levels of air pollution. This heterogeneity in study design further makes it difficult to compare both across studies and study populations. Third, air pollution measurement sites varied markedly in their geographical location and distribution. This could create potential confounding in observations, as population density and urban/rural sites, and elevation were found to be potential modifying factors. While we sought to identify these effect modifiers, future research performing meta-analyses of specific air pollutants could attempt to address this potential issue. Our analysis also did not attempt to take into account the impact of alternative measurements on pollutants’ concentration, since these studies have been performed elsewhere, although we did restrict our analysis to those studies using validated measurement techniques.

Notwithstanding these limitations, our analysis has several important strengths. First, to our knowledge, this is the only study to perform a comprehensive review of the relation between COVID-19 lockdowns in Europe and North America and the major air pollutants established as harmful to human health. Second, while we could not perform a meta-analysis due to heterogeneity in studies, we nonetheless were able to evaluate a large quantity of studies and ascertain common trends and factors.

Future research is needed to understand the extent to which air pollution reductions were sustained as harmful human activity resumed.

Our findings have powerful implications for policy. Worldwide, countries are struggling to find optimal ways to reduce anthropogenic driven air pollution. Air pollution poses both substantial risks to human health, including its recently established role in increasing COVID-19 transmissibility. Our review of the unique historical case of COVID-19 lockdowns reveals the tremendous potential for reducing harmful air pollution through limiting human activities, especially on road transit and industrial emissions. While we would of course not recommend further lockdowns to improve air quality, it does raise challenging questions about the potential sustainability of human activities and directly establishes their causality in driving pollution.

## Supplementary data

Supplementary data are available at *EURPUB* online.

*Conflicts of interest*: None declared.

Key points

- COVID-19 lockdowns may have contributed to lower air pollution levels.
- NO_x_, CO_x_ and PM levels fell substantially in association with COVID-19 lockdowns.
- In contrast, ozone tended to increase, as nitrous oxide levels dropped.
- Greater reductions occurred when lockdowns were more severe.
- Human activity has a profound and avoidable effect on air pollution levels.

## Supplementary Material

ckac118_Supplementary_Data
