# Supplementary material for: The impact of COVID-19 lockdown on air pollution in Europe and North America: a systematic review
Source: Eur J Public Health. 2022 Sep 8;32(6):962–8. doi: 10.1093/eurpub/ckac118 (PMC9494388; doi:10.1093/eurpub/ckac118)
Supplement: ckac118_Supplementary_Data [file ckac118_supplementary_data.zip › ejph-2022-05-om-0244-File006.docx]

Appendix S1. Known harmful and suspected effects of the Environmental Protection Agency’s Criteria and Hazardous Air Pollutants

| Pollutants | Sources | Health effects |
| --- | --- | --- |
| *Environmental Protection Agency Criteria Air Pollutants: Known to be harmful* |  |  |
| NO2 (Nitrogen Dioxide) | Road traffic, mostly vehicles with diesel engines; fossil fuel combustion; power plants | Asthma and reduced lung function; Irritation of eyes, nose and throat; breathing problems; Impacts on liver, spleen and blood |
| CO (Carbon Monoxide) | Traffic; household heating | Is a harmful gas in the atmosphere; it affects the environment and human health heavily |
| SO2 (Sulfur Dioxide) | The majority of SO2 in air is of human origin such as power plants, heating systems and some industrial processes. Also, volcanoes emit SO2 to the atmosphere | Headache and anxiety; cardiovascular diseases |
| Ground-Level Ozone (O3) | Ozone is formed by chemical reactions which react with the sunlight, as well as with pollutants emitted into the air from transportation, landfills, household chemicals and natural gas extraction. Also causes the decrease of NOx, NO and increases insolation and temperatures.  Causes the decrease of NOx in a VOCs-limited environments (Volatile Organic Compounds), a decrease of NO (Nitrogen Oxide) or an unusual increase of insolation and temperatures.  Is formed by chemical reactions (triggered by sunlight) involving pollutants emitted into the air, including those by transport, natural gas extraction, landfills and household chemicals | Leading risk factor for asthma and the worsening of lung function, especially in people with COPD. Irritation of eyes, nose and throat; breathing problems; cardiovascular diseases |
| Particular Matter  PM1, PM2.5, PM5, PM10 | Particulate matter (PM) are particles that are suspended in the air. Primary PM emissions result from: power generation, domestic heating, vehicle engines and also from non-exhaust emissions from brakes, tyres, read wear and dust resuspension. Secondary PM is formed: NH3, NOx, SO2, non-methane volatile organic compounds and natural sources (desert dust, wildfires). The combustion of solid and liquid fuels, such as power generation, domestic heating and in vehicle engines. Road transport emissions also include non-exhaust emissions from brake, tyre and road wear and road dust resuspension. PM also forms in the air from secondary PM precursors, including ammonia, nitrogen oxides, sulphur dioxides and non-methane volatile organic compounds. Natural sources result from the transport of desert dust and wildfires | Impacts on the central nervous system; chronic obstructive pulmonary disease; lung cancer; irritation of eyes, nose and throat; breathing problems; cardiovascular diseases; impacts on the reproductive system |
| Lead | Ore and metals processing, aircrafts which use leaded aviation fuel, waste incinerators, utilities, and lead-acid battery manufacturers | Accumulation in bones; Affects the nervous system, kidneys, immune system, reproductive and developmental systems and the cardiovascular system; Also affects the oxygen carrying capacity of the blood. Infants and young children are especially sensitive to lead exposures, which may contribute to behavioral problems, learning deficits and lowered IQ |
| *Environmental Protection Agency Hazardous Pollutant Known or Suspected Carcinogens* |  |  |
| C6H6(Benzene) | Motor vehicle exhaust, burning coal and oil, gasoline service stations | Acute exposure can cause: drowsiness, dizziness, headaches, eye, skin, and respiratory tract irritation; At high levels can cause unconsciousness; Chronic exposure: can cause blood disorders (reduced numbers of red blood cells and aplastic anemia), reproductive effects, increased incidence of leukemia EPA has classified benzene as known human carcinogen for all routes of exposure |
| PAHs (Polycyclic aromatic hydrocarbon) levels | PAHs occur in coal, crude oil, and gasoline and are produced when coail, oil, wood, gas, garbage and tobacco are burned. Also, when we cook food i.e. meat in high temperatures PAHs are formed Naphthalene is a PAH that is produced commercially in the United States to make other chemicals and mothballs | The most significant endpoint of PAH toxicity is cancer; Increased incidences of lung, skin, and bladder cancers are associated with occupational exposure to PAHs |
| NOx (Nitrogen Oxides) | High motor vehicle traffic; building heating;  industrial processes | NO_x_ along with NO2 react with other chemicals in the air and form PM and O3which are harmful to the respiratory system when inhaled |
| Black Carbon (BC) | Traffic; household heating; vehicular and biomass burning emissions; combustion of carbonaceous fossil fuels; biomass and vegetation | Respiratory and cardiovascular effects and premature death |
| VOCs (Volatile Organic Compounds) Common VOCs include acetone, benzene, ethylene glycol, formaldehyde, methylene chloride, perchloroethylene, toluene and xylene | Vehicular traffic; Gasoline; Diesel emissions; Wood burning; Oil and gas extraction and processing; Industrial emissions; other incomplete combustion processes | Irritation of eyes, nose and throat; Breathing difficulty and nausea; Damage of central nervous system and other organs; cancer; (Not all VOCs have all the above health effects) |

*Sources:* European Environment Agency: https://www.eea.europa.eu/

Environmental Protection Agency: https://www.epa.gov/
