# Supplementary material for: The impact of COVID-19 lockdown on air pollution in Europe and North America: a systematic review
Source: Eur J Public Health. 2022 Sep 8;32(6):962–8. doi: 10.1093/eurpub/ckac118 (PMC9494388; doi:10.1093/eurpub/ckac118)
Supplement: ckac118_Supplementary_Data [file ckac118_supplementary_data.zip › ejph-2022-05-om-0244-File007.docx]

Appendix S2. Search Strategy

*Final PubMed Search 7th June 2021: 589*

(((2020:2021[pdat]) AND (lock* OR restrictive measures OR isolation OR self-isolation)) AND (COVID-19 OR SARS-CoV-2 OR 2019-nCoV OR n-CoV OR coronavirus OR "SARS-CoV-2"[Mesh] OR "COVID-19"[Mesh])) AND (Air pollut* OR Particulate matter OR PM OR PM2.5 OR PM10 OR Nitrogen dioxide OR ozone OR sulfur dioxide OR carbon monoxide)

*Final WoS Search: 7th June 2021: 656*

ALL=((lock* OR restrictive measures OR isolation OR self-isolation) AND (COVID-19 OR SARS-CoV-2 OR 2019-nCoV OR n-CoV OR coronavirus) AND (Air pollut* OR Particulate matter OR PM OR PM2.5 OR PM10 OR Nitrogen dioxide OR ozone OR sulfur dioxide OR carbon monoxide))

*NOTES:* **LANGUAGE:** (English) Years 2020 and 2021 options ticked
