# Supplementary material for: The impact of COVID-19 lockdown on air pollution in Europe and North America: a systematic review
Source: Eur J Public Health. 2022 Sep 8;32(6):962–8. doi: 10.1093/eurpub/ckac118 (PMC9494388; doi:10.1093/eurpub/ckac118)
Supplement: ckac118_Supplementary_Data [file ckac118_supplementary_data.zip › ejph-2022-05-om-0244-File008.docx]

**Appendix S3**

**Summary of study findings by geographic region, red = negative, blue = positive, grey = mixed / no effect**

*Europe:* *67 studies and 247 estimated pollutant changes*

*
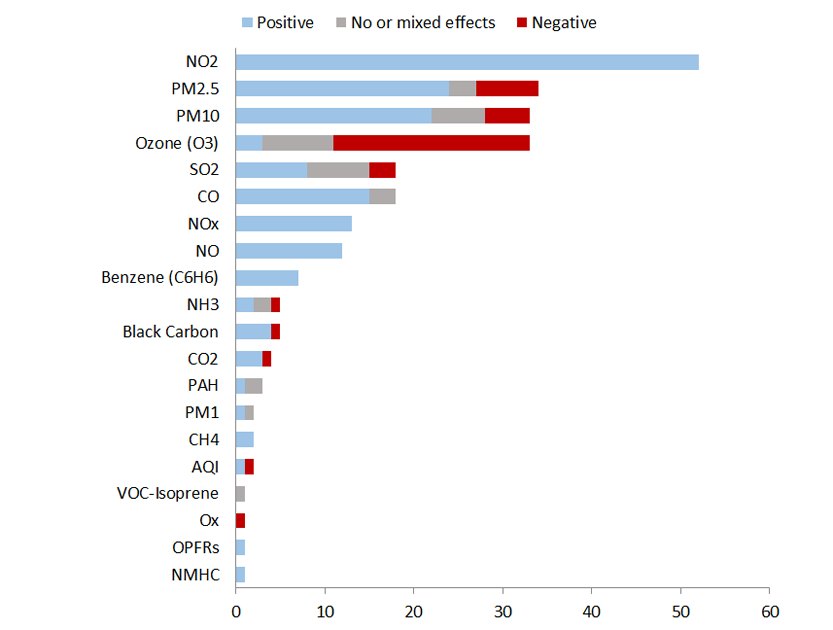
*

*North America: 18 studies and 53 estimated pollutant changes*

*
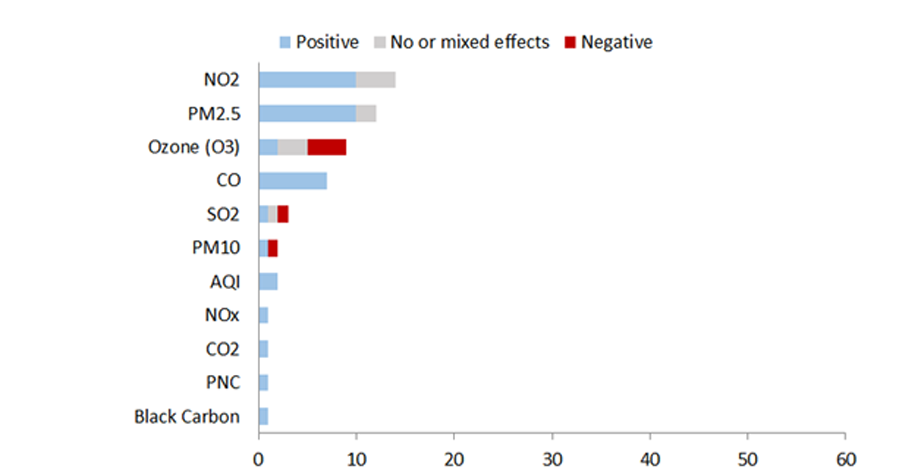
*

Note: AQI (Air Quality Index), PAH (Polycyclic aromatic hydrocarbons), VOC (Volatile Organic Compounds), PNC (Particle Number Concentration), OPFRs (Organophosphate Flame Retardants), NMHC (Non-methane Hydrocarbons)
