# Supplementary material for: The impact of COVID-19 lockdown on air pollution in Europe and North America: a systematic review
Source: Eur J Public Health. 2022 Sep 8;32(6):962–8. doi: 10.1093/eurpub/ckac118 (PMC9494388; doi:10.1093/eurpub/ckac118)
Supplement: ckac118_Supplementary_Data [file ckac118_supplementary_data.zip › ejph-2022-05-om-0244-File009.docx]

**Appendix S4 includes the following tables:**

**Table 1. Summary Table of Included Studies from Europe**

**Table 2. Summary Table of Included Studies from North America**

**Table 3. Summary Table of Included Studies from Multi Countries**

**Table 1. Summary Table of Included Studies from Europe**

| **Study** | **Country** | **Geographic Setting** | **Time Period** | **Baseline / Reference Period** | **Air Pollution Outcome** | **Main Findings** |
| --- | --- | --- | --- | --- | --- | --- |
| Achebak et al.^1^  2021 | Spain | 47 cities in Spain and the Balearic Islands | March 15-June 21, 2020 | 2010-8 | NO2, O3 | NO2 changed by -51% and -36.4%. O3 changed by -1.1% and 0.6% (-12.4 to 23.0%), during the lockdown (57 days) and deconfinement (42 days) periods, respectively. |
| Altuwayjiri et al.^2^  2020 | Italy | Two sites: Milano – via and a suburban residential area Bareggio | January -June 2020 /April 11 – June 3, 2020 | 2019 | PM2.5, Black Carbon (BC), NO2, and Benzene (C6H6) mass concentrations (PAHs, levoglucosan, etc.) | The concentrations of the examined pollutants declined considerably (p<0.0001) PM2.5 and BC declined ~78% and ~90% from January to May, respectively. NO2 declined by ~58% from the Pre-Pandemic period (51.94±8.00 μg/m3) to the lockdown period (21.44±12.05 μg/m3). |
| Aydin et al.^3^  2020 | Turkey | 20 different regions in Turkey | December 2019- May 2020. April 2020 (during lockdown), May 2020 (reopening of some businesses). | December 2019 (pre-pandemic) | PM2.5, O3, Air Quality Index (AQI) | PM2.5 had substantial reductions in the majority of the cities, although O3 increased. Highly polluted regions like Erzurum had substantial improvements in air quality, by over 2.5-fold. Air quality improved from an unhealthy to a moderate. |
| Baldasano^4^,  2020 | Spain | 24 air quality stations in Madrid and 9 in Barcelona, Spain | March 2020 | 2018- 2019 | NO2 | Madrid and Barcelona showed a significant decrease by about 75%. |
| Bassani et al.^5^  2021 | Italy | 6 stations in Rome | 1st March -30 April, 2020 | 2019 | NO2, NO, O3, CO | There were clear declining trends in NO and NO2 levels due to the reduced traffic and industrial emissions during the lockdown period..The reduction ranged from − 34 to − 76% and from − 30 to − 65% for NO and NO2, respectively. CO levels declined less compared to those of NO and NO2.O3 levels showed a slight average increase in urban and suburban background stations and a slight average decrease in rural background stations. |
| Brancher^6^,  2021 | Austria | 17 monitors in  Vienna | January -September 2020 (lockdown in Austria: March 16- April 13, 2020) | 2015-2019 | NO2, O3, Ox | NO2 levels declined by average -20.1% [13.7-30.4%] during lockdown whereas O3 increased by +8.5%. |
| Briz-Redon et al.^7^  2020 | Spain | Barcelona, Bilbao, Lleida, Madrid, Pamplona, Santander, Santiago de Compostela, Sevilla, Valencia, Vigo, Zaragoza | March 15-March 29, 2020 and March 30-April 12, 2020 | 2019 | CO, SO2, PM10, O3, NO2 | Generally the pollutant levels, especially NO2 declined in 2020. The rest of the pollutants showed decreased levels in many cities, with certain increases. SO2, CO and PM10 reduced only in some cities. O3 increased in some cities during lockdown, associated with the decreased NO2 and CO levels. |
| Brown et al.^8^  2021 | United Kingdom | Schools within 0.5 km of NO2 monitoring sites in England | 23 March-23 April 2020 | 2015-9 | NO2 | NO2 levels reduced during lockdown compared to the pre-pandemic period by 8.53 (μg/m3), or 35.13%, and 15.74 (μg/m3) or 40.82%, at background and traffic sites. |
| Celik and Gul^9^,  2020 | Turkey | 19 air monitoring stations (AMSs)in European and Asian side of Istanbul | March 1- May 22, 2020 | 2019 | PM10, SO2, CO, NO2, NO, NOx, O3 | PM10, NO2, NO, and NOx levels declined during the pandemic compared to the pre-pandemic period. SO2 and CO concentrations showed a non-uniform trend in different AMSs. |
| Collivignarelli et al.^10^  2020 | Italy | 22 meteorological control units in Milan | Partial Lockdown (March 9, 2020 -March 22, 2020), and Total Lockdown (March 23- April 5, 2020). | February 7 – February 20, 2020 | PM10, PM2.5, BC, benzene, CO, SO2, NO2, NOx, O3, NH3 | Movement restrictions resulted in a significant reduction of pollutant levels mainly due to vehicular traffic: PM10, PM2.5, BC, benzene, CO and NOx. CO reduction was particularly noticeable in the city of Milan where vehicle traffic in the main source of these emissions.  SO2 reduced during lockdown only in the city of Milan while it did not change in the Sub-area A and Sub-area B. During total lockdown there was a significant decline in NO2 and a significant increase in O3 due to minor NO concentration. |
| Davidovic et al.^11^  2021 | Serbia | 4 locations in Novi Sad | 1 February- 30 April 2020 | Six weeks before and six weeks during lockdown | PM10, PM2.5, SO2, NO2, NO, NOx, CO, O3 | Particulate matter andSO2 levels, did not change significantly NO2, NO, and NOx showed lower levels during most hours of the day, due to restrictions on vehicular movement. CO showed a lower level during night. O3 increased during the day showing maximal levels from 10 to 16 h with a median of about 70 g/m3 during the week before and 80 g/m3 during the first 10 days of the lockdown. |
| De Maria et al.^12^  2021 | Italy | 4 urban traffic stations in Apulia Region (Bari, Brindisi, Lecce, Taranto) and 2 urban background stations (Foggia and Barletta) | March-April 2020 | 2019 | NO2, PM10 | The mean NO2 concentration levels showed a decrease and there was no change in PM10 levels. |
| Dobson and Semple^13^, 2020 | United Kingdom | 70 PM2.5 and 89 NO2 monitoring stations in Scotland | 24 March- 23 April 2020 | 2017-2019 | PM2.5, NO2 | NO2 concentrations remained close to constant in 2017, 2018 and 2019 but decreased significantly in 2020. PM2.5 levels remained almost at the same levels in 2017 but decreased in 2018 and 2019. |
| Donateo et al.^14^  2021 | Italy | The site is located inside the university campus, about 3.5 km southwest of the town of Lecce in the southeast of Italy. | January 1- July 31 2020  (Pre-lockdown phase: January 1 - March 10; Lockdown phase: March 11-May 3; Post Lockdown period: May 4-July 31 | 2016–2019 | PM10, PM2.5 | Comparing the lockdown phase and the mirror period in 2016–2019 for PM concentrations, a decrease has been observed. A reduction of 14% in PM10, from a value of 25 (±2 μg m−3) to 22 (±2 μg m−3) during the 2020 lockdown. PM2.5 mass concentration changed from 13.6 (±0.5 μg m−3) to 11.7 (±0.8 μg m−3), with a percentage reduction of 14%. |
| Donzelli et al.^15^  2021 | Spain | All air quality monitoring stations in Valencia city | 1 January-30 September 2020;  Pre-lockdown: 1 January-14 March;); Lockdown:15 March-17 May; Phase 1: 18 May-31 May; Phase 2: June-14 June; Phase 3:15 June -20 June; Post lockdown: 21 June- 30 September | 2019 | PM10,PM2.5, NO2, NO, NOx, and O3 | During lockdown PM levels were notably different compared to the previous year in different areas. The highest PM10 and PM2.5 reduced levels levels were reported at València Centre, València Avd Francia, and València Pista de Silla (all of the urban traffic type) where there was a reduction of 58%–42%, 56%–53%, and 60%–41% respectively. In all monitoring stations, NOx, NO2, and NO concentrations decreased. O3 levels declined during the lockdown period and this was related to weather conditions. There was no significant variability of meteorological conditions in 2019 and 2020. |
| Donzelli et al.^16^  2020 (The Effect of the Covid-19 Lockdown on Air Quality in Three Italian Medium-Sized Cities) | Italy | 11 air-monitoring stations (4 stations in Florence, 2 stations in Pisa and 3 stations in Lucca) | 1 January to 12 August 2020;[Pre-lockdown: 1 January–8 March; [Lockdown: 9 March–3 June]; [Post-lockdown: 4 June–12 August]; | 2019 | PM10, PM2.5, NO2, O3 | PM reduction was not associated with lockdown restrictions in urban centers, except in areas with heavy traffic. There was a significant decline in NO2 concentrations in all AMSs . O3 levels did not change during the lockdown period. |
| Dragic et al.^17^  2021 | Serbia | 7 air quality measurement stations in Novi Sad | March 15–May 6, 2020 | 2018-2019 | SO2, NO2, O3, PM10, PM2.5 | PM2.5, NO2, PM10 and SO2 daily concentrations reduced by 35%, 34%, 23% and 18%, respectively. However, O3 daily concentrations increased by 8%, despite the reduction of the primary precursors. |
| Filonchyk et al.^18^  2020 | Poland | 28 air quality monitoring stations located in urban, suburban, rural and industrial in Warsaw, Wroclaw, Lodz, Krakow and Gdansk | March- May 2020 | 2018-2019 | PM2.5, PM10, SO2, NO2, AOD | AOD reduced in April and May by approximately -23% and-18% compared to 2018–2019. The reductions for PM2.5 in April and May were from -11.1% to -26.4% and from-8.7 to -21.1% respectively. PM10 reduced from - 8.6% to - 33.9% and from - 8.5% to -31.5% compared to 2019. AOD data showed reductions of aerosol concentration in the air column in April and May approximately by - 23% and - 18% compared to 2018 and 2019. Tropospheric NO2 showed a clear reduction from 15 March - 25 April approximately by - 10 to - 19%. |
| Gama et al.^19^  2020 | Portugal | 24 monitoring stations in the mainland Portugal | Lockdown or partial lockdown days (16 March-31 May, 2020) | 2015-2019 | NO2, PM10 | The mean reduction levels were higher for NO2 (41%) than for PM10 (18%). Between 1 January -15 March and 16 March -31 May, PM10 had an average reduction of approximately 7 μg/m3 (corresponding to 30%) , while NO2 mean difference was about 12 μg/m3, representing a reduction of 55%. |
| Granella et al.^20^  2021 | Italy | 83 monitoring stations in Lombardy | January to early May 2020 (lockdown:22 February - 4 May) | 2016–2019 | PM2.5, NO2 | PM2.5 reduced by 3.84 μg m−3 (16%) and NO2 by 10.85 μg m−3 (33%). |
| Grivas et al.^21^  2020 | Greece | Athens | 23 March–10 May 2020 (lockdown) | 2016-2019 | NO2, CO, CO2, BC and components, PM2.5 | All investigated pollutants declined significantly during lockdown (p<0.05). Urban CO2 had a remarkable reduction by 53% and PM2.5 by 18%.  Significant reductions were reported when compared the 2020 lockdown period with past years, However, levels rebounded instantly after the lockdown period. |
| Gualtieri et al.^22^  2020 | Italy | 58 air quality and meteorological stations in Milan, Bologna, Florence, Rome, Naples, and Palermo | 24/02/2020 -30/04/2020 | 2019 | NO2, O3, PM2.5, PM10 | NO2 levels dropped significantly in all urban areas (from 24.9% in Milan to 59.1% in Naples).On the contrary, O3 remained unchanged or even increased (up to 13.7% in Palermo and 14.7% in Rome), possibly due to the reduced O3 titration triggered by lower NO and NOx emissions. |
| Hicks et al.^23^  2021 | United Kingdom | Marylebone Road (Roadside), Honor Oak Park (Background) and Egham (Background) monitoring sites in London | 23 March (day of lockdown) - 30 June 2020 | 2019 | PM10, PM2.5, CO2, NOx | PM10 and PM2.5 reduced by 49% and 76%, respectively. CO2 reduced at the start of the UK lockdown on 23 March 2020. |
| Higham et al.^24^  2020 | United Kingdom | London, Glasgow, Belfast, Birmingham, Manchester, Liverpool | 23 March (day of lockdown) -30 June 2020-100 days following lockdown | 2019 and 7-year average (2013-2019) | NO2, O3, SO2, PM2.5, PM10 | NOx levels across the country dropped substantially (∼50%) and PM2.5 dropped (∼ 20%). O3 levels increased (∼ 10%), and SO2 levels more than doubled across the country. Compared to the previous 7 years, UK-wide SO2 levels are more than double. Meteorological conditions could be a potential factor for this increase, but other factors should be studied too. |
| Hormann et al.^25^  2020 | Austria | Graz, 2 sites in areas with heavy traffic, 2 in commercial and industrial areas and 1 in a residential area. | February 1 -June 14; Phase 0: Feb 1–Mar 15; Phase 1: Mar 16–Apr 14;  Phase 2: Apr 15–May 18; Phase 3: May 19–June 14 | 2015–2019 | NO2, NO, CO, PM10 | NO2 and NO showed a rapid decline due to the decrease of traffic. During Phase 1 of the lockdown, the mean NO2 levels declined by 35-41%. PM10 and CO mostly exhibited little change. |
| Ikhlasse et al.^26^  2021 | France | 629 stations across French regions | January 2020 -July 2020 | pre/during and post‑  lockdown periods comparison 2020 | SO2, O3, NO2, NOx, CO, C6H6, PM2.5, PM10 | All daily maximum pollutant levels decreased during containment phase, except from O3. O3 increased by 27.19% during lockdown and continued growing by 21.35% as well during post lockdown period. In different French regions, daily concentrations declined by 18.18%, 37.14%, 20.36%, 9.28%, 44.38%, 5.1% and 44.38%, respectively, for SO2, NO2, CO, C6H6, NOx, PM2.5 and PM10. However, decreased levels of other pollutants were not sustained after deconfinement for NO2, NOx and PM10. |
| Jakovljevic et al.^27^  2020 | Croatia | Two measuring sites: urban residential and urban traffic in Zagreb | March–May 2020 | 2019 | PM1, polycyclic aromatic hydrocarbons (PAHs), NO2 | NO2 and PM1 particles decreased by 35% and PAHs by 26% at the traffic measuring site. At the residential measuring site, only NO2 levels declined slightly.PM1 particles and PAHs were similar to the previous year. |
| Jephcote et al.^28^  2020 | United Kingdom | 129 monitoring stations | 30/03/2020 to 03/05/2020 (weeks 14-18) | 2017-2019 | NO2, O3,PM2.5 | There was a 69% reduction in traffic overall (74% reduction in light and 35% in heavy vehicles) during lockdown. NO2 reduced by 38.3% ( -8.8 mg/m3) and PM2.5 by 16.5% ( -2.2 mg/m3). In contrast, O3 increased by 7.6% (+4.8 mg/ m3). |
| Kaskaoutis et al.^29^  2021 | Greece | Athens (Thisio urban area), Finokalia (Crete) | pre-lockdown:1–22 March 2020;lockdown: 23 March–3 May 2020; post-lockdown:4–31 May 2020 | 2016-2019 | spectral-scattering (bsca) and absorption (babs) coefficients, black carbon (BC) concentrations, single-scattering albedo (SSA), scattering and absorption Ångström exponents (SAE,AAE) | During lockdown babs showed the highest decrease compared to the pre-lockdown (-39%) and to the same period in previous years (-36%). This was more intense during morning traffic hours (-60%), due to the large decrease in vehicle emissions. AAE increased during lockdown due to reduced emissions from fossil-fuel combustion. Bsca decreased by -21% whereas SAE and SSA increased slightly by 6%. |
| Kazakos et al.^30^  2021 | United Kingdom | 98 monitoring sites for NO2 and 21 monitoring sites for PM2.5 in London | 23 March- 23 April 2020 | 2017–2019 | NO2, PM2.5 | After lockdown applied, NO2 and PM2.5 levels dropped by 40.9% ± 6% for NO2 and 13.9% ± 4% for PM2.5. |
| Koukouli et al.^31^  2021 | Greece | Athens, Thessaloniki, Larisa, Volos, Patras, Heraklion | March–April 2020 | 2019 | NO2 | NO2 monthly mean observations showed a change of between -34% and +20% and between-39% and +5% with an average of -15% and -11% for March and April 2020 respectively, in comparison with the previous year. |
| Lee et al.^32^  2020 | United Kingdom | 66 urban traffic and 62 urban background sites across the UK | 23 March–31 May 2020 | 2015–2019 | NO2, O3 | NO2 declined by an average of 48% at urban traffic and by 40% at urban background. O3 increased by 11% at urban background sites and by 48% at three urban traffic sites. |
| Lonati and Riva^33^,  2021 | Italy | Fixed monitoring sites distributed all over the territory of Po Valley(Emilia-Romagna, Lombardia, Piemonte, and Veneto) | First semester of 2020 | 2014-2019 | NO2, benzene, ammonia (NH3) | Nitrogen oxides and benzene emissions from road traffic reduced by 35-40% compared to the previous years. Higher reductions, approximately 50% were observed at high-volume-traffic sites in urban areas. On the contrary, NH3 levels due to agriculture sector emissions did not show any changes even at high-volume-traffic sites in urban areas. |
| Lovarelli et al.^34^  2020 | Italy | 14 data stations at Lombardy region (Brescia, Cremona, Lodi, and Mantua) | January–March 2020 | 2016–2019 | NH3, PM2.5, NOx | PM2.5 decreased by 19%–32% in 2016–2019 and by 21%–41% in 2020. NOx levels were lower in 2020 than in 2016–2019 (reduction in March respect to February of 22–42% for 2016–2019 and of 43–62% for 2020). NH3 emissions showed no reduction due to livestock and agriculture activities. |
| Lovric et al.^35^  2020 | Austria | 5 measurement sites in Graz | 3rd January 2020-10th March 2020, a lockdown set, Lockdown(LD) (10th March 2020 - 2nd May 2020) and a hard lockdown set (HLD) (20th March 2020-14th April 2020) | 2014-2019 | PM10, NO2, Ox, O3 | During lockdown, NO2 and PM10 reduced by -36.9 to 41.6%, and 6.6 to 14.2%, respectively. O3 increased by 11.6-33.8%.. |
| Malpede and Percoco^36^,  2021 | Italy | 71 Italian provinces | February 24- May 4, 2020 | 2016–2019 | PM10, PM2.5, NO2 | Lockdown reduced PM10 and NO2 levels by 17–18%, while the effect on PM2.5 remains unclear. |
| Marinello et al.^37^  2020 | Italy | 9 stations in Reggio Emilia | 1 February—29 May 2020 covering the lockdown period 10 March—18 May 2020 | 2019 | PM10, PM2.5, O3, NO2, CO | NO2 decline was 32% in correspondence with the AQMS classified as “traffic” and 41% at the “urban background” station. The decrease was more significant during lockdown: -41% and -52% for the traffic and urban background station, respectively. CO reduced by -22%, while PM10 and PM2.5 concentrations in 2020 increased compared to 2019.PM10 increased by 27% at the traffic station and by 23% at the urban background station, while PM2.5 had a growth of 31% at the urban background station. O3 levels increased due to the reduction of circulating traffic. |
| Mehlig et al.^38^  2021 | United Kingdom | United Kingdom | Pre-pandemic restrictions: 1 January-22 March; 1st national lockdown: 23 March-18 June; Local measures: 19 June-4 November; 2nd national lockdown: 5 November-1 December; Local measures: 2 December-31 December; Pandemic period: 23 March-31 December | 2017–19 | CO2, NOx, PM2.5 | Large reductions took place during the two lockdowns: up to 22% for CO2, 47% for NOx, and 29% for PM2.5. |
| Mesas-Carrascosa et al.^39^  2020 | Spain | 11 cities with more than 275000 inhabitants(Madrid, Valencia, Barcelona, Sevilla, Malaga, Cordoba, Alicante, Palma, Zaragoza, Valladolid, Bilbao) | 1st January- 30th April 2020 | 2019 | NO2 | High levels of NO2 were reported in the city of Madrid, which has the largest number of inhabitants in Spain, as well as in other cities with a large number of inhabitants. The relationship between the population density map in Spain and the NO2 distribution followed the same pattern before lockdown. NO2 was mainly related to vehicle traffic The comparison between the NO2 concentration values before and after the lockdown measures shows a strong relationship with the number of inhabitants. Population’s activity level and the reduction of NO2 values are significantly correlated. |
| Munir et al.^40^  2021 | United Kingdom | Leeds, Sheffield, Manchester | Pre-lockdown: 1 February 2020–23 March 2020; lockdown:24 March 2020–10 May 2020; Post-lockdown: 11 May 2020–30 June 2020 | 2019 | NO, NO2, NOx, PM10, PM2.5 | Reductions in NO (56.68–74.16%), NO2 (18.06–47.15%), and NOx (35.81–56.52%).However, PM10 and PM2.5 levels increased during lockdown with ranges from 21.96–62.00% and 36.24–80.31%, respectively. |
| Orak and Ozdemir^41^,  2021 | Turkey | Public air quality monitoring network in 81 cities in Turkey | January-November 2020 (first mobility restrictions March 2020) | 2015–2019 | PM10, SO2 | PM10 and SO2 concentrations were lower in 67% and 59% of the cities, respectively in April 2020 compared to the previous five years (2015–2019). |
| Ozbay and Koc^42^,  2021 | Turkey | 4 stations in Izmit | April-May 2020 (23 lockdown days and constituted a partial lockdown period in whole) | 2019 | PM10, PM2.5, NOx, SO2, O3 | PM10, PM2.5, and NOx decreased remarkably in the partial lockdown period in all the stations. The most significant NOx reduction was observed in Station 2 in the city center with a ratio of 58.26%, whereas the highest reduction ratios for PM10 and PM2.5 (36.25% and 39.03%, respectively) were recorded in Station 1. O3 concentrations increased in both Stations 1 and 4. SO2 concentrations also increased in the studied period. |
| Petetin et al.^43^  2020 | Spain | 50 Spanish provinces over the mainland and individual islands over the Balearic and Canary Island:38 provinces with urban background stations and 37 provinces with traffic stations | 14 March to 23 April 2020; phase I: 14–29 March 2020, phase II: 30 March–9 April 2020, phase III: 10–23 April 2020 | 2017–2019 | NO2 | 50% decrease in NO2 concentration levels on average over all provinces and islands. |
| Piccoli et al.^44^  2020 | Italy | Lombardy | February 24 till end of March 2020 | Pre-lockdown: 1 January 7 March for years 2014- 2020;  Lockdown: 8 March -30 April for years 2014- 2020. | NO2 | NO2 decreased progressively as traffic reduced. Decrease in NO2 concentration reflects progressively reduced traffic contraction. When there is 71% traffic reduction the NO2 levels decline by one third. |
| Potts et al.^45^  2021 | United Kingdom | London and Manchester for NO2 and across UK for PM2.5 and ozone. | 23 March- 31 May 2020 | 2019 | NO2, PM2.5, O3 | NOx decreased by ∼20% during the lockdown nationwide In western areas of the country the reduction was 22-23%, 29% in the southeast and more than 40% in London. Nationally, PM2.5 reduced by 1.1 μg m−3, ranging from 0.6 μg m−3 in Scotland to 2 μg m−3 in the southwest, whereas O3 increased slightly by 0.24ppbv. There was an increase by 0.89ppbv and 1.0ppbv for Manchester and London, respectively. In rempte and rural areas O3 decreased. |
| Prats et al.^46^  2020 | Spain | Barcelona(urban site) and Aigüestortes (continental backgroud in remote high mountains, in the Pyrenees) | B1:15 October 2019–9 January 2020(86 days); B2:9 January 2020–15 July 2020(188 days). Lockdown: March 15–June 22 2020 (100 days) | 2018-2019 | CO, PM10, NO, NO2, O3  Polycyclic aromatic hydrocarbons (PAHs), polychlorobiphenyls (PCBs), hexachlorobenzene (HCB), pentachlorobenzene (PeCB), and organophosphate flame retardants (OPFRs); | All of the investigated pollutants decreased during lockdown. The following decreased significantly: CO, NO, NO2, and PM10 (between -28 and -76%) and O3 increased (+45%) during lockdown. Generally, PAHs had the higher reduction from -68 to -87%. Especially benz[a]anthracene and pyrene decreased by -87 and -81%, respectively. PeCB showed a remarkable decrease by -90.5%, followed by -79% for HCB. PCBs declined by -37 to -69%. |
| Querol et al.^47^  2021 | Spain | 11 metropolitan areas: 8/10 most populated metropolitan areas in Spain (Madrid; Barcelona; València; Sevilla;Málaga; Bilbao; Zaragoza; Murcia; and three less populated areas (A Coruña; Valladolid; Badajoz;) | Pre-pandemic:  14 Feb – 15 Mar; lockdown:  16 Mar – 30 May;  relaxation:  31 May – 31 Jul; | 2015-2019 | PM2.5, PM10 (before and after North African dust outbreak PM2.5sub; PM10sub); NO, NO2, CO, SO2, NH3, O3 | NO2 dropped under 50% of the WHO annual air quality guidelines (WHOAQGs), while PM2.5 decreased less than expected because of lower traffic and increased contributions from agricultural and domestic biomass burning as well as, meteorological conditions. O3 decreased in rural areas during the relaxation period (june-July) due to -20% reduced mobility but in urban areas there were heterogeneous results associated with the period and the location. After neutralizing the meteorology effect O3 declined in 5/11 cities. The rest 6 cities showed no reductions or increases. CO reduced to 100–428 μg/m3 for all cities and SO2 decreased too. NH3 levels were only available for VAL and BIL and were reduced by 9% and 38% respectively during lockdown in BIL. |
| Rodrigo-Comino and Senciales-Gonzales^48^,  2021 | Spain | Barcelona, Madrid, Málaga, Sevilla, Valencia | March - May 2020 | 2009-2019 | O3, NO2, SO2, CO, PM10, PM2.5 | Slight reduction of pollutant levels in highly populated cities of Spain. For O3, there was no increase of the average values reached in the past years in March, April and May. NO2 decreased in all cities during the restriction period and especially in April, except from Madrid. SO2 levels varied during the lockdown and the year 2020. The available data for PM2.5, PM10 and CO was not possible to be compared in all cities and years. |
| Ropkins and Tate^49^,  2021 | United Kingdom | 153 stations for NO, NO2, NOx and 75 for O3 | 01 January -30 June 2020 | 01 January 2015 -31 December 2019. | NO, NO2, NOx, O3, PM10, PM2.5 | NO, NO2 and NOx decreased (on average) 32-50 % at roadsides on lockdown. O3 levels increased by (on average) 20% on lockdown. Across the UK, increases were observed for both PM10 and PM2.5 during lockdown(PM10 5.9 μg m−3 to 6.3 μg m−3 and PM2.5 3.9 μg m−3 to 5.0 μg m−3). |
| Rossi et al.^50^  2020 | Italy | Two monitoring stations in Padova | 8 March -30 April 2020 | 2017- 2018 | NO, NO2, NOx, PM10 | NO, NO2, and NOx reduced significantly in both stations. NO decreased by 46% and 54% in Station A and Station B, respectively. NO2 declined by 34% in Station A and 40% in Station B. NOx reduced by 36% in Station A and 44% in Station B. PM10 levels reduced by 2% in Station A and 10% in Station B. |
| Rosu et al.^51^  2021 | Romania | 4 air quality monitoring stations in Galati | January- June 2020; lockdown: 23 March 2020–15 May 2020 | 2019 | NO2 | NO2 levels were smaller during lockdown in 2/4 stations, mostly during workdays. NO2 reduced approximately by 10% probably due to the reduced traffic. |
| Ruberti and Romano^52^, 2020 | Italy | Greater Salento region-Taranto:11 active monitoring stations; Lecce: 8 ARPA units, and Brindisi:16 functioning stations; | 8 weeks of lockdown period (10March-3 May 2020); 6 previous weeks (27 January-9 March); the last period (4 May-14 June) | 2019 | NO2 | NO2 concentrations decreased by−23.2% compared to previous year, during the lockdown period. |
| Rugani and Caro^53^,  2020 | Italy | Italy | 1 March-30 April 2020 | 2015-2019 | Carbon Footprint (CO2) | Carbon Footprint in the lockdown period is ~−20% lower than the mean Carbon Footprint calculated during the past. |
| Sahraei et al.^54^  2021 | Turkey | Ankara, Istanbul | January - May 2020 | 2019 | AQI | Significant improvement of air quality during lockdown. Ankara and Istanbul improved by 9% and 47%, respectively. |
| Salma et al.^55^  2020 | Hungary | Budapest | Five periods in 2020 were compared: i) 1 January- - 11 March (Pre-emergency phase). ii)11 March-27 March (pre-restriction phase). iii) 27 March-17 May (Restriction of movement phase). iv) 17 May- 17 June (Post-restriction phase). v)17 June-31 July (Post-emergency phase) | 2017–2019 | NO, NO2, CO, O3, SO2, Particulate Matter (PM) | The highest changes took place during the severest restriction (28 March-17 May 2020). NO, NO2, CO, total particle number (N6–1000) and particles with a diameter <100 nm reduced during 2020 by 68 %, 46 %, 27 %, 24% and 28 %, respectively, compared to the average for the years 2017–2019. O3 showed an increasing tendency. |
| Sannino et al.^56^  2021 | Italy | Naples, 4 stations | March 13–April 30, 2020 | 2019 | C6H6 (Benzene), CO, NO2 , SO2, PM10, PM2.5, PM1 | NO2 decreased significantly by 49–62% in urban and green suburban area, while CO and SO2 reduced remarkably in urban or industrial districts of the city (50–58% and 70%, respectively). PM at ground level declined by 29–49%. Benzene showed a reducing tendency. |
| Sbai et al.^57^  2021 | France | 79 monitoring stations in Auvergne-Rhône-Alpes region | March 17-May 11, 2020 | 2019 | NO2, NO, PM10, PM2.5, O3, VOC(volatile organic component), CO, SO2, and isoprene | In Lyon NO2, NO, and CO levels reduced by 67%, 78%, and 62%, respectively, resulting in 80% decrease in road traffic. However, O3, PM10, and PM2.5 increased by 105%, 23%, and 53%, respectively, during the lockdown. Volatile organic component (VOC), isoprene and SO2 remain almost stable. |
| Sifakis et al.^58^  2021 | Greece | Rethymno, Crete | Lockdown period, 2020 | 2018 - 2019 | CO2, CO, NOx, CH4 | Significant changes monitored in three out of four examined air pollutants for the year 2020, compared to the past two years (2018 & 2019). There is a decrease in the rest of the pollutants than the 2018 mean levels, by 66%, 61%, and 75% for CO, NOx, and CH4, respectively. CO2 levels were slightly increased in 2020 by 1%. |
| Tobias et al.^59^  2020 | Spain | Barcelona | March 14-March 30, 2020 | 2019 | PM10, SO2, NO2,O3, Black Carbon | Black Carbon and NO2 which are related to traffic emissions reduced (−45 to −51%). PM10 showed lower reduction (−28 to −31.0%). On the contrary, O3 increased (+33 to +57% of the 8 h daily maxima), probably due to lower titration of O3 by NO and the decrease of NOx in a VOC-limited environment. Low SO2 levels (around 1.0 to 2.6 μg/m3 as averages of the different sites) in the study period and the slight changes (−0.2 and +0.1 μg/m3, for UB and TR sites, close to the detection limit) cannot define an evidenced trend. |
| Varotsos et al.^60^  2021 | Greece | Athens, Thessaloniki, Volos, Larisa | March 11 (start of restrictive measures)- May 11 (first day of the partial withdrawal of the restrictive measures) | 2013-2019 | NO2, O3, PM10, PM2.5 | NO2 reduced levels during lockdown do not indicate a clear step-by-step or rapid reduction that could be expected after the abrupt application of shutdown. There is no clear indication that surface O3 levels have declined during. No significant change in the PM10 concentration was observed during the lockdown period. PM2.5 in some representative monitoring stations in the Athens did not show a significant decline. In Thessaloniki, NO2,O3, PM10, and PM2.5 levels did not show any particular differences during the lockdown period. PM10 and PM2.5 levels in Volos and Larisa stations showed no statistical difference between the values collected during the lockdown period and their long- term mean. |
| Velders et al.^61^  2021 | The Netherlands | 9 rural background stations, 11 urban background stations, and 14 traffic stations | 16 March-10 May 2020 | 2017-2019 | Nitrogen oxides (NOx and NO2), particulate matter (PM10 and PM2.5), O3 | NOx and NO2 levels during 2020 were mostly below those in 2017–2019 already before the lockdown period, at the rural and urban background and traffic locations. O3 levels are higher than those in 2017–2019. PM2.5 and PM10 showed lower concentrations in February and beginning of March than in 2017–2019. NOx concentrations at traffic locations reduced significantly, on average, by 39%, with a 95% confidence interval (CI) of 31–47%. The reduction at urban background locations is 33% (95% CI 25–40%) and at rural background locations is 20% (95% CI 11–29%). NO2 levels declined slightly on average 30% (95% CI 25–35%), 26% (95% CI 21–32%), and 18% (95% CI 10–25%) for traffic, urban, and rural background locations, respectively. |
| Viatte et al.^62^  2021 | France | Paris | 17 March–1 June (17 March start of lockdown) | 2015–2019 | Ammonia (NH3), NO2 and PM2.5 | NO2 decreased by 24% (averaged over 17 March–1 June, compared to 1 January–17 March 2020). NO2 levels during 2020 lockdown were at their lowest compared to the previous 5 years. Simultaneous increases of PM2.5 and NH3 hourly concentrations were reported during most of pollution episodes. PM2.5 increased by 16% after the beginning of lockdown. |
| Viteri et al.^63^  2020 | Spain | Ground-air quality monitoring networks in Madrid, Albacete, Puertollano, San Pablo de los montes | lockdown and the de-escalation period (14 March - 30 June) | Previous months (January- February 2020) and March-June 2013-2019 | NOx, O3, SO2, CO, PM2.5, PM10, BTXs (Benzene, Toluene and Xylenes),NMCH (non-methane hydrocarbons),  CH4, NH3 | NOx, CO, BTXs, NMHC and NH3 reduced significantly. PM showed lower changes during the crisis whereas O3 did not show clear trends. Pollutant concentrations remained below pre-lockdown levels despite the gradual de-escalation. SO2 decrease compared to the previous 5 years was negligible. |
| Vultaggio et al.^64^  2020 | Italy | Urban area of Palermo | 1 January 2020- 31 July 2020 and lockdown period (10 March-30 April) | 2015–2019 | CO, NO2, O3, PM10 | Pollutants linked to vehicular traffic reduced sharply during lockdown. From 10 March to 30 April, CO, NO2, and PM10 concentrations decreased by approximately 51%, 50%, and 45%, respectively. O3 concentration was increased. |
| Wyche at al.^65^  2020 | United Kingdom | 25 automatic monitoring stations in the South-East UK | January-May (Lockdown start: 23/3/2020) | 2015–2019 | NO2,O3, PM10, PM2.5 | NO2 decreased during the lockdown whereas O3 increased. PM10 and PM2.5 levels peaked with respect to the 2020 average, during the regional pollution episodes. Thus there is limited evidence of the PM reduced levels which can be attributed to lockdown. |

**Table 2. Summary Table of Included Studies from North America**

| **Study** | **Country/State** | **Geographic Setting** | **Time Period** | **Baseline / Reference Period** | **Air Pollution Outcome** | **Main Findings** |
| --- | --- | --- | --- | --- | --- | --- |
| Al-Abadleh et al.^66^  2021 | Canada | 16 ground sites in Southern Ontario | Apr-Jun 2020 | 2017-19 | NO_2,_ CO, O3 and PM2.5 | NΟ2 and CO dropped on average by 20%; only 2-3/16 sites witnessed significant drops in 03 and PM2.5 |
| Brodeur et al.^67^  2021 | USA | County level monitoring-3 nearest air quality monitoring stations | 1 January -30 June 2020 | 1990-2016 | PM2.5, AQI | Particulate matter concentration levels reduced during the safer-at-home period by 25%. State orders significantly reduce Air Quality Index. |
| Chadwick et al.^68^  2021 | USA | 71 unique locations in Salt Lake County | 11 February-10 May 2020 | 2019 | PM2.5 | PM2.5 monthly concentrations decreased significantly by 71.1% and 21.3% from 2019 to 2020 for the periods from March 11-April 10 and April 11-May 10, respectively. Greatest reductions were seen at lower elevation, more urbanized areas, probably due to the already low PM2.5 levels at higher elevations and more residential areas. |
| Chen et al.^69^  2020 | USA | 28 long-term air quality stations across the U.S. | March - April 2020 | 2017-2019 | NO2, CO, O3, PM2.5, PM10 | NO2 reduced by 49% and CO by 37%. The reductions were statistically significant at 2/3 of the sites and increased with the population density. PM2.5 and PM10 declined most in Northeast and California/Nevada metropolises where NO2 decline most! O3 were mixed and minor. |
| El-Sayed et al.^70^  2021 | USA | 30 monitoring stations in six major cities across the state of Florida | Pre-lockdown: 15 February - 15 March 15 2020 Lockdown: 15 March 15-April 2020 | 2015-2019 | PM2.5, O3, NO2, SO2, CO | 3 main factors identified: vehicular emissions, power generation and pollution transport. NO2, CO, and O3 levels decreased in most cities and were attributed to restrictions in mobility and the decrease in vehicle usage amid the lockdown. SO2 increased probably due to increased power generation and was positively correlated with PM2.5. PM2.5 did not change compared with historical levels amid lockdown apart from Jacksonville (increase). |
| Elshorbany et al.^71^  2021 | USA | States with high traffic volume: New York, Illinois, Florida, Texas, and California | March - May 2020 | 2015–2019 | NO2, CO, O3 | CO and NO2 decreased due to lockdown and decrease in traffic volume. O3 declined related to the decrease or increase of NO2 levels, dur to O3 photochemical sensitivity. |
| Ghosal and Saha^72^,  2021 | USA | 48 core-based statistical areas (CBSA) across the United States | 1 January–29 June 2020 | Pre-lockdown and lockdown period in 2020 | PM2.5 | PM2.5 decreased during lockdown in densely populated areas such New York and Baltimore. However, the opposite happened in the Los-Angeles-Long Beach-Anaheim region, one of the most populated and polluted regions in USA. |
| Griffin et al.^73^  2020 | Canada | Southern Ontario | Pre-lockdown: 16 February–15 March 2020,lockdown: 16 March–8 May 2020 | 2019 | NO2 | NO2 reduced by approximately 60% during lockdown compared to the previous month. About 25% of the aforementioned reduction is due to meteorological and seasonal changes.  Due to the lockdown NO2 declined by over 40%. These changes vary spatially, and in certain locations columns declined by over 50%. During 2019, there was 0–2% decline over the Greater Toronto Area, which is expected since there were no emission declines in spring 2019. |
| Hudda et al.^74^  2020 | USA | Somerville | March-May 2020 (The lockdown period is defined as March 24, 2020) | The Somerville route: 2018–2019 and the I-93 route:  2012–2013 | Ultrafine particle number concentration [PNC] and black carbon [BC] | Median PNC and BC contributions from traffic were 60–68% and 22–46% lower, respectively, during the lockdown compared to pre-pandemic conditions, and corresponding reductions in total on-road concentrations were 45-69% and 22-56%, respectively. On I-93 route traffic was 41% lower and the median PNC was 60% lower compared to 2012-2013. |
| Jia et al.^75^  2020 | USA | Memphis Metropolitan Area | 25 March- 4 May 2020 | 2017-2019 | PM2.5,NO2, O3 | The grand regional mean concentrations of PM2.5, NO2, and O3 were 7.5±2.6 g/m3, 16.5±9.4 ppb, and 44.5±8.4 ppb, respectively, during the baseline period, and 7.8±2.5 g/m3, 18.6±10.4 ppb, and 42.6±7.44 ppb, respectively, during the lockdown period. No immediate air pollution drops occurred during the lockdown, in comparison to the pollution levels in the prior month. PM2.5 levels increased by 23%, 21%, and 19% at Ncore, Hernando, and Marion stations, respectively. NO2 concentration did not change at Marion. O3 concentrations increased by 29–36% during the lockdown. |
| Liu et al.^76^  2020 | USA | California | Lockdown: 19 March–7 May 2020 | 2015-2019 | NO2, O3, CO, PM10, PM2.5 | California shows a 38%, 49%, and 31% reduction in NO2, CO and PM2.5 levels during lockdown compared to before (January 26–March 18) in 2020. These are 16%, 25% and 19% sharper than the means of the previous five years, respectively. |
| Mashayekhi et al.^77^  2021 | Canada | Toronto, Montreal, Vancouver, and Calgary | 22 March–2 May 2020 | 2010-2019 | NO2, PM2.5, O3 | NO2 levels decreased during lockdown. For PM2.5,Montreal was the only city with a higher-than-usual seasonal decline, whereas for O3 all four cities remained within the previous decadal range. |
| Naeger and Murphy^78^,  2020 | USA | California: Los Angeles, Fresno, Bakersfield, San Francisco | March - April 2020 | 2019 | NO2, PM2.5 | NO2 reduced by approximately 35% in Los Angeles and Fresno and by 25% in San Francisco and Bakersfield compared to 2019, which led to PM2.5 decline and improved air quality conditions at the surface. |
| Parker at al.^79^  2020 | USA | South Coast Air Basin, California | March - June 2020 | 2015-2019 | O3, NO2, NOx, PM2.5 | PM2.5 and NOx levels reduced across the basin due to up to 50% decreases in traffic during lockdown. O3 declined in the western part of the basin and increased in the downwind areas. The NOx decline in 2020 (approximately 27% basin-wide) is in addition to ongoing declines over the last two decades. |
| Shehzad et al.^80^  2021 | USA | NYC, Brooklyn, Manhattan, Queens, and Staten Island County | 1 January-31 May 2020 | Pre/during lockdown comparison (AQI compared to 2018) | PM2.5, CO,NO2, SO2, O3, AQI | PM2.5 levels reduced significantly from 10.3 to 4.0 μg/m3 and NO2 levels decreased by up to 52% in 1st phase of lockdown. O3 levels increased by 44.4%. Brooklyn, Manhattan, Queens, and Staten Island County encountered 18.75%, 55.62%, 47.14%, and 47% decrease in Air Quality Index due to lockdown as compared to 2018, respectively. CO levels decreased to 0.18 ppm in Bronx and New York County, representing a 74.2% reduction due to lockdown. In New York NO2 levels declined by 52% on March 23, 2020, compared to March 09, 2020. PM2.5 was 10.3 μg and 8.0 μg on March 09, 2020, and March 17, 2020, respectively, though, as the lockdown announced in New York, the concentration /m3 level of PM2.5 reduced to 4.0 μg on March 23, 2020. |
| Straka et al.^81^  2020 | USA | Los Angeles, California, Chicago, Illinois, Washington DC | Stay at home measures were implemented on 12^th^ (Chicago), 19^th^ (Los Angeles), and 20^th^(Washington DC) of March 2020 | 2019 | NO2, PM2.5 | Both NO2 and PM2.5 concentration were reduced in LA, Chicago, and DC during March–April. The largest reduction of NO2 was about 66% in LA, whereas Chicago saw the largest reduction in PM2.5. |
| Tian et al.^82^  2021 | Canada | Vancouver, Edmonton, Saskatoon, Winnipeg, Toronto , Halifax, St. John's | February -September 2020 (Considering declaration of emergency on different days) | 2018-2019 | CO2,SO2, NO2, CO | CO2 emissions reduced significantly in April in 2020 due tolockdown. NO2 decreased in 2020 compared with the same period in 2019-2018 except for Toronto and Winnipeg. The most significant change was in Edmonton with a 78.6% decline from February to July 2020. CO levels decreased in Vancouver, Halifax and Edmonton much faster. SO2 concentration changes in each city were not significant. |
| Wong et al.^83^  2021 | USA | 56 stations in 54 cities across the US | February- May 2020 | 2014-2020 | NO2 | Half of the NO2 measurements showed a 23.08% and 30.38% reduction of the NO2 levels before lockdown. A considerable reduction of NO2 after lockdown was seen in most cities, while an unexpected, minor increase was found in four cities. |

**Table 3. Summary Table of Included Studies from Multi Countries**

| **Study** | **Country** | **Geographic Setting** | **Time Period** | **Baseline / Reference Period** | **Air Pollution Outcome** | **Main Findings** |
| --- | --- | --- | --- | --- | --- | --- |
| Acharya et al.^84^  2020 | Multi-country | Europe | Lockdown periods (2020) | 2017–2019 | AOD, NO2, SO2 | AOD: 20% reduction in central and eastern Europe; 30-60% increase in western Europe such as France, Spain, and Portugal; 20% drop over majority of areas in the US. NO2: reductions in Europe of about 20–40% except for eastern and northern European countries. Similar figures observed for the US. SO2 increased by 30% in south-east and eastern European countries, whereas SO2 decreased by up to 10% in the majority of the north and western European countries, including the southern part of England. SO2 increased by 10-30% over the majority of areas in the USA. |
| Bauwens et al.^85^  2020 | Multi-country | Italy, Spain, France, Germany | February–April 2020 | 2019 | NO2 | NO2 levels were unusually low in Italy. NO2 decreased by 30% in Spain and France during lockdown. In Germany and Belgium NO2 decrease was more moderate (-20%), possibly because the restrictions were less strict in these countries. |
| Chossiere et al.^86^  2021 | Multi-country | France, Italy, Spain, Germany, United Kingdom, Norway, Switzerland | Lockdown periods in each country 2020 | 1 January 2019- 7 July 2020 | PM2.5, O3, NO2 | The average NO2 levels between the start of the local lockdown and 6 July change was -24% in Europe. In contrast, in Europe PM2.5 levels did not change with tighter lockdown restrictions. A +8.5% change in PM2.5 was found in Europe during the period of study and only 2% of the European population had statistically significant changes in PM2.5. The change in O3 levels in Europe was -3.1% and was not significant. |
| Collivignarelli et al.^87^  2020 | Multi-country | UK, Italy, France, 52 air quality control units | Lockdown periods in 2020, London: 26 March-12 May, Milan: 11 March- 3 May, Paris: 17 March- 10 May | 2017-2019 | NO2 | NO2 decreased significantly due to traffic. London: 71.1 % - 80.8 %; Milan: 8.6 % - 42.4 %; Paris: 65.7 % - 79.8 %. . |
| Connerton et al.^88^  2020 | Multi- country | France (Paris), USA  (Los Angeles  and New York) | March 2020, including the first days of the month, when no restrictive measures were in place yet | 2015-2019 | CO,O3,PM2.5,NO2 | CO decreased by 24% in Los Angeles, 19% in New York and 67% in Paris, while NO2 decreased by 38% in Los Angeles, 24% in New York and 39% in Paris. PM2.5 declined by 37%, 24% and 28% in Los Angeles, New York and Paris, respectively. O3 increased in New York and Paris but decreased in Los Angeles. |
| Fu et al.^89^  2020 | Multi-country | Istanbul (Turkey),Rome (Italy), Madrid (Spain), Paris (France), London (UK), Berlin (Germany), Moscow (Russia) | March -June 2020 (with differences according to the state) | 2017- 2019 | AQI, NO2, SO2, CO, ground-level O3, PM2.5, PM10 | NO2 reduced for all cities during the lockdown period Compared to the previous years. The SO2 decreased significantly in Moscow and Madrid With the highest decrease (-54.8%). SO2 increased significantly in Istanbul (+29.3%) and Berlin (+29.5%). CO decreased significantly in London (-53.5%) compared to 2019. O3 increased significantly in Paris (+26.8%) and London (+48.0%), and significantly decreased in Istanbul (-43.6%), relative to 2019. However, the AQI level in 2020 is statistically insignificant in comparison to 2018. PM2.5 decreased in all cities, except in Rome with an increase of +8.3% during the lockdown period relative to 2019 and the 2017–2019.PM10 decreased in all European cities. |
| Goldberg et al.^90^  2020 | Multi-country | 20 cities in USA and Canada: San Jose, Los Angeles, Toronto, Philadelphia, Denver, Atlanta, Detroit, Boston, Washington, Montreal, New York City, New Orleans, Las Vegas, Houston, Chicago, Phoenix, Austin, Dallas, Miami, Minneapolis | 15 March- 30 April 2020 period is the most stringent lockdown in North America | Comparison: before (1 January -29 February 2020) and after (15 March -30 April 2020); 2019; | NO2 | NO2 reduced by 9.2%-43.4% among 20 cities in North America, with a median of 21.6%. The highest decreases (>30%) were in San Jose, Los Angeles, and Toronto, and smallest (<12%) were in Miami, Minneapolis, and Dallas. |
| Gope et al.^91^  2021 | Multi-country | UK, Spain, Italy (London, Madrid, Rome) | April 2020 | 2019 | AQI, PM2.5, PM10, O3, NO2, CO, SO2 | All three cities recorded improvements in overall AQI and decreases in CO. PM10 dropped in London and Madrid but increased in Rome by about 25%. Similarly, ozone increased in London and Madrid but in Rome it declined by 10%. Only in Madrid did SO2 drop, by about 50 NO2 has drastically decreased in all the displayed places. PM2.5 reduced and only in Rome increased in the lockdown period due to the burning of forests. |
| He et al.^92^  2021 | Multi-country | Italy, Spain, France, UK | 1 January-30 June 2020 | 2015–2019 | PM2.5, O3, NO2 | PM2.5 and NO2 decreased globally by 16.1% and 45.7%, respectively. In contrast, O3 increased by 5.4% globally. PM2.5 and NO2 levels decreased over all continents, with a maximum reduction of 20.4% for PM2.5 in East Asia and 42.5% for NO2 in Europe. |
| Kumari and Toshniwal^93^,  2020 | Multi-country | London-UK(20 stations), Madrid-Spain(4 stations), Rome-Italy(10 stations | 1January - 10 June 2020;  23/03/2020-1/06/2020(London), 14/03/2020-On going(Madrid), 11/03/2020-18/05/2020(Rome);  March, April, May 2020 | 2019 | PM2.5, PM10, NO2, SO2 O3 | PM2.5, PM10 and NO2, reduced remarkably during lockdown. PM2.5 levels dropped by 20.2-34.3% and PM10 reduced by 23.7- 47.3%. However, NO2 has a higher decline by 31.6-64.5%. SO2 levels showed a mixed trend during the lockdown phase. |
| Liu et al.^94^  2021 | Multi- country | UK, USA | UK lockdown: 22 March – 14 May 2020, USA lockdown: 21 March – 14 June 2020 | Peri/pre/post lockdown period for 2020, 2019 and 2010-2019 | NO2 | NO2 reduced in most countries due to the shutdown of non-essential industries and reductions in transportation. After these policies were lifted, NO2 emissions gradually rebounded to normal levels. |
| Liu et al.^95^  2020 | Multi-country | EU, UK, USA, Russia | 1 January-30 June 2020 | 2019 | CO2 | In the first half year of 2020, the most pronounced decrease took place in U.S. ( −13.3%), followed by EU27 & UK ( −12.7%), with substantial but progressively decreases in Russia (−5.3%). |
| Rodríguez-Urrego and Rodríguez-Urrego^96^,  2020 | Multi-country | Bosnia & Hersegovina, North Macedonia, Kosovo, Bulgaria, Poland, Serbia, Romania, Turkey, Israel, Lithuania, Nicosia, Czech Republic, Slovakia, Hungary, France, Austria, Belgium, Ukraine, Switzerland, United Kingdom, Germany, Portugal | Lockdown start: March-April 2020 | One month before and after the start of Quarantine | PM2.5 | PM2.5 levels decreased at an average of 23% in half of the European capitals. However, in the other half, there was an increase in the confinement season. For example Budapest, which has an annual average concentration of 48 mg/m3, is a city where PM2.5 increased by 35%. |
| Sahraei et al.^97^  2021 | Multi-country | UK, Sweden, Germany, France, Spain, Italy, Turkey, USA, Canada | Lockdown periods between 15 January  -31 May 2020 | 2019 | PM2.5, PM10, SO2, CO, O3, NO2 | During lockdown the percentages of PM2.5, PM10, SO2, CO, and NO2 were decreased by 16%, 21%, 41%, 48%, and 35% lower than those in the same time in 2019. O3 levels declined compared to the previous year. |
| Sannigrahi et al.^98^  2021 | Multi-country | Chicago, Denver, Detroit, Los Angeles, New York, and Philadelphia, Antwerp, Barcelona, Brussels, Cologne, Frankfurt, London, Madrid, Milan, Paris, Rotterdam,Turin, Utrecht. | 1 February- 11 May 2020 | 2019 | NO2, PM2.5, PM10 | NO2 levels reduced most significantly (>-40%) in Brussels and Paris, followed by −35% to −40% in Barcelona and London, −25% to −35% in Rotterdam, Antwerp, Madrid, Utrecht, −15%–25% in Milan, Frankfurt, Detroit, −5%–15% in New York, Denver, Chicago, and less than −5% in Los Angeles, respectively. PM2.5 and PM10 decreased in cities. The highest reduction (>35%) in PM2.5 was recorded for London, Rotterdam, and Brussels, followed by 25%–35% reduction in Antwerp, Frankfurt, Utrecht, 15%– 25% reduction in Denver, Paris, New York, 5%–15% reduction in Detroit, Chicago), and <5% reduction in Madrid and Philadelphia, respectively. However, PM2.5 increased in Los Angeles and Milan, while PM10 declined most over the European cities, with ranges >35% (London), 25%– 35% (Paris, Frankfurt), 15%–25% (Brussels, Denver, Rotterdam, Antwerp, Utrecht, Barcelona, Chicago), 5%–15% (Detroit, Madrid, Philadelphia). PM10 levels increased in Milan and Los Angeles. |
| Shakoor et al.^99^  2020 | Multi-country | USA (California, Florida, Louisiana, North Carolina, and Alabama) | 19 March - 30 April 2020 | 2019 | CO, NO2, SO2, PM2.5, PM10 | CO, NO2 and PM2.5 decreased by 19.28%, 36.7% and 1.10%, respectively, while PM10 and SO2 increased by 27.81% and3.81% respectively in five selected states of the USA during the lockdown period |
| Shi et al.^100^  2021 | Multi-country | Milan and Rome in Italy, Madrid in Spain, London in United Kingdom, Paris in France, Berlin in Germany | Tightened restrictive measures were implemented from: 23 January 2020 in northern Italy, 14 March 2020 in Spain, 17 March 2020 in France, 22 March 2020 in Germany,  23 March 2020 in the United Kingdom | December 2015- May 2020 | PM2.5, PM10, O3, NO2, CO, and SO2 and other auxiliary pollutants NO and NOx | NO2 decreases were not as large as expected, at reductions of 10-50%. O3 increased by 2-30% (except for London) and PM2.5 decreased in most cities studied but increased in London and Paris. PM10 levels were similar PM2.5 with a slight difference in some cities. The deweathered CO decreased substantially, ~40% lower than that during the same period in 2018. The deweathered SO2 concentration in 2020 is much lower than that in 2018. In London, deweathered SO2 declined for a few days before the lockdown began at roadside sites. Deweathered SO2 in Rome decreased about a month before the lockdowns but did not change during the lockdowns. In New York, a decline in deweathered SO2 is observed a week after lockdown began. |
| Sicard et al.^101^  2020 | Multi-country | France-Nice, Italy-Rome and Turin, Spain -Valencia | 1January -18 April 2020 | 2017–2019 | NO, NO2, PM 2.5, O3, PM 10 | NO2 mean concentrations reduced in all European cities, about 53% and 65% at urban and traffic stations, respectively. NO declined further by 63% and 78% at urban and traffic stations in Europe, respectively. PM2.5 and PM10 at urban stations had smaller reductions in Europe (~8%). The NOx declined on average by 49% in all cities. O3 levels were about 10% higher in Southern Europe. |
| Singh et al.^102^  2021 | Multi-country | Russia, France, Spain, United Kingdom, USA | Phase-1: 25 January  - 31 January 2020  Phase-2 (1^st^ wave, extensive lockdowns in many countries): 25 May -31 May 2020); and Phase-3 (2nd wave, less restrictive interventions):25 October-31 October 2020. | 2019 | CO, NO2, SO2,O3 | CO levels decreased in all countries, though with significant spatial variation. NO2 levels also decreased generally in some countries. Conversely, in USA and Russia, regional concentrations of NO2 and O3 increased significantly, in some case by more than 50% during the “lockdown”. In France, Spain, UK, USA and Russia, O3 levels increased in 2019 compared to previous years. They slightly decreased in 2020 especially during the lockdown. SO2 increased in France and the USA influenced by local factors in almost all cases |
| Skiriene and Stasiskiene^103^,  2021 | Multi-country | United Kingdom, Spain, France, Sweden, Northern Italy region | Pre-lockdown (1January- 29- February 2020), I period (March–April 2020), when national lockdowns were announced, and II period (May 2020) when countries decided to apply less restrictive regulations | 2018-2019 | PM2.5, PM10, NO2 | During the I period, NO2, PM2.5, PM10 decreased in all countries. The average concentration of PM2.5 and PM10 changes indicate the impact of factors other than lower transport flows, industrial, and economic activities. In Northern Italy, the national lockdown did not affect the average concentration reduction of PM2.5 and PM10 in Pre-lockdown, I and II periods and they were higher by 24.1% and 20.9% during the II period of 2020, compared to the same period in 2019 |
| Solberg et al.^104^  2021 | Multi-country | All EU countries with EEA monitors | March–July 2020 | 2015–2019 | NO2 | NO2 reduced by 60% in Spain, followed by 51% in Italy, 51% in France, 47% in Portugal and 43% in Great Britain. The least impact was in Poland (22%) and Hungary (23%). |
| Steinbrecht et al.^105^  2021 | Multi-country | Norway, Finland, Greenland, Sweden, United Kingdom, Germany, Poland, Netherlands, Ireland, Belgium, Switzerland, France, Spain | April - August 2020 | 2000-2020 | O3 | O3 (from 1-8 km altitude) was on average 7% (≈4 nmol/mol) below the 2000–2020 climatological mean. Such low O3has not been observed in any previous year since at least 2000. |
| Venter et al.^106^  2020 | Multi-country | Austria, Belgium, Croatia, Denmark, Finland, France, Germany, Hungary, Ireland, Italy, Luxembourg, Mexico, Netherlands, Norway, Poland, Portugal, Serbia, Slovakia, Spain, Sweden, Switzerland, United Kingdom | January - May 2020 | 2017-2019 | NO2, PM2.5, O3 | In 34 countries, NO2 decreased by 60% (95% CI 48 to 72%), and fine particulate matter by 31% (95% CI: 17 to 45%), whereas O3 increased slightly by 4% (95% CI: −2 to 10 during lockdown |
| Virghileanu et al.^107^  2020 | Multi-country | Europe | 15 January - 30 April 2020 | 2019 | NO2 | Remarkable NO2 decreases even of 85% in some of the European big cities |
| Wang et al.^108^  2021 | Multi-country | USA, Europe (Italy, Germany, France, UK, Russia) | February, March, April, October, November 2020 | 2019 | NO2 | Europe and America are major contributors in the reductions of the global NO2, approximately 15.7% and 8.8%, respectively. |
| Wang and Li^109^,  2021 | Multi-country | Italy (Milan), Spain (Madrid), United Kingdom (London),  New York ( USA) | Beginning of the lockdown to October 30, 2020 | 2017–2019 | NO2, PM2.5, O3, SO2 | NO2 had the highest decrease during 2020 lockdown, followed by PM2.5. O3 increased slowly due to the photochemical chain reaction caused by the decline of NO2. SO2 experienced minor changes which were not obvious. Specifically, compared with the average concentration in 2020 with the same periods from 2017 to 2019, NO2 in 2020 decreased by 40–50 %, PM2.5 decreased by 10–30 %, O3 increased by 17–20 % and SO2 increased slightly. |

**References**

1 Achebak H, Petetin H, Quijal-Zamorano M, Bowdalo D, Pérez García-Pando C, Ballester J. Trade-offs between short-term mortality attributable to NO2 and O3 changes during the COVID-19 lockdown across major Spanish cities. *Environmental Pollution* 2021;286:117220.

2 Altuwayjiri A, Soleimanian E, Moroni S, et al. The impact of stay-home policies during Coronavirus-19 pandemic on the chemical and toxicological characteristics of ambient PM2.5 in the metropolitan area of Milan, Italy. *Science of The Total Environment* 2021;758:143582.

3 Aydin S, Nakiyingi BA, Esmen C, Guneysu S, Ejjada M. Environmental impact of coronavirus (COVID-19) from Turkish perceptive. *Environment Development And Sustainability* 2021;23:7573–80.

4 Baldasano JM. COVID-19 lockdown effects on air quality by NO2 in the cities of Barcelona and Madrid (Spain). *Science of The Total Environment* 2020;741:140353.

5 Bassani C, Vichi F, Esposito G, Montagnoli M, Giusto M, Ianniello A. Nitrogen dioxide reductions from satellite and surface observations during COVID-19 mitigation in Rome (Italy). *Environmental Science and Pollution Research* 2021;28:22981–4.

6 Brancher M. Increased ozone pollution alongside reduced nitrogen dioxide concentrations during Vienna’s first COVID-19 lockdown: Significance for air quality management. *Environmental Pollution* 2021;284:117153.

7 Briz-Redón Á, Belenguer-Sapiña C, Serrano-Aroca Á. Changes in air pollution during COVID-19 lockdown in Spain: A multi-city study. *Journal of Environmental Sciences* 2021;101:16–26.

8 Brown L, Barnes J, Hayes E. Traffic-related air pollution reduction at UK schools during the Covid-19 lockdown. *Science of The Total Environment* 2021;780:146651.

9 Celik E, Gul M. How Covid-19 pandemic and partial lockdown decisions affect air quality of a city? The case of Istanbul, Turkey. *Environment, Development and Sustainability* 2021;24:1616–54.

10 Collivignarelli MC, Abbà A, Bertanza G, Pedrazzani R, Ricciardi P, Carnevale Miino M. Lockdown for CoViD-2019 in Milan: What are the effects on air quality? *Science of The Total Environment* 2020;732:139280.

11 Davidović M, Dmitrašinović S, Jovanović M, Radonić J, Jovašević‐stojanović M. Diurnal, Temporal and Spatial Variations of Main Air Pollutants Before and during Emergency Lockdown in the City of Novi Sad (Serbia). *Applied Sciences* 2021;11:1212.

12 De Maria L, Caputi A, Tafuri S, et al. Health, Transport and the Environment: The Impacts of the COVID-19 Lockdown on Air Pollution. *Frontiers in Public Health* 2021;9:354.

13 Dobson R, Semple S. Changes in outdoor air pollution due to COVID-19 lockdowns differ by pollutant: evidence from Scotland. *Occupational and Environmental Medicine* 2020;77:798–800.

14 Donateo A, Dinoi A, Pappaccogli G. Impact on Ultrafine Particles Concentration and Turbulent Fluxes of SARS-CoV-2 Lockdown in a Suburban Area in Italy. *Atmosphere*  2021;12:407.

15 Donzelli G, Cioni L, Cancellieri M, Llopis‐morales A, Morales‐suárez‐varela M. Relations between Air Quality and Covid-19 Lockdown Measures in Valencia, Spain. *International Journal of Environmental Research and Public Health* 2021;18:2296.

16 Donzelli G, Cioni L, Cancellieri M, Morales AL, Suárez-Varela MMM. The Effect of the Covid-19 Lockdown on Air Quality in Three Italian Medium-Sized Cities. *Atmosphere* 2020;11:1118.

17 Dragic N, Bijelovic S, Jevtic M, Velicki R, Radic I. Short-term health effects of air quality changes during the COVID‑19 pandemic in the City of Novi Sad, the Republic of Serbia. *International journal of occupational medicine and environmental health* 2021;34:223–37.

18 Filonchyk M, Hurynovich V, Yan H. Impact of Covid-19 lockdown on air quality in the Poland, Eastern Europe. *Environmental Research* 2021;198:110454.

19 Gama C, Relvas H, Lopes M, Monteiro A. The impact of COVID-19 on air quality levels in Portugal: A way to assess traffic contribution. *Environmental Research* 2021;193:110515.

20 Granella F, Reis LA, Bosetti V, Tavoni M. COVID-19 lockdown only partially alleviates health impacts of air pollution in Northern Italy. *Environmental Research Letters* 2021;16:035012.

21 Grivas G, Athanasopoulou E, Kakouri A, et al. Integrating in situ Measurements and City Scale Modelling to Assess the COVID–19 Lockdown Effects on Emissions and Air Quality in Athens, Greece. *Atmosphere*  2020;11:1174.

22 Gualtieri G, Brilli L, Carotenuto F, Vagnoli C, Zaldei A, Gioli B. Quantifying road traffic impact on air quality in urban areas: A Covid19-induced lockdown analysis in Italy. *Environmental Pollution* 2020;267:115682.

23 Hicks W, Beevers S, Tremper AH, et al. Quantification of Non-Exhaust Particulate Matter Traffic Emissions and the Impact of COVID-19 Lockdown at London Marylebone Road. *Atmosphere*  2021;12:190.

24 Higham JE, Ramírez CA, Green MA, Morse AP. UK COVID-19 lockdown: 100 days of air pollution reduction? *Air Quality, Atmosphere and Health* 2021;14:325–32.

25 Hörmann S, Jammoul F, Kuenzer T, Stadlober E. Separating the impact of gradual lockdown measures on air pollutants from seasonal variability. *Atmospheric Pollution Research* 2021;12:84–92.

26 Ikhlasse H, Benjamin D, Vincent C, Hicham M. Environmental impacts of pre/during and post-lockdown periods on prominent air pollutants in France. *Environment, Development and Sustainability* 2021;23:14140–61.

27 Jakovljević I, Štrukil ZS, Godec R, Davila S, Pehnec G. Influence of lockdown caused by the COVID-19 pandemic on air pollution and carcinogenic content of particulate matter observed in Croatia. *Air Quality, Atmosphere and Health* 2021;14:467–72.

28 Jephcote C, Hansell AL, Adams K, Gulliver J. Changes in air quality during COVID-19 ‘lockdown’ in the United Kingdom. *Environmental Pollution* 2021;272:116011.

29 Kaskaoutis DG, Grivas G, Liakakou E, et al. Assessment of the COVID-19 Lockdown Effects on Spectral Aerosol Scattering and Absorption Properties in Athens, Greece. *Atmosphere*  2021;12:231.

30 Kazakos V, Taylor J, Luo Z. Impact of COVID-19 lockdown on NO 2 and PM 2.5 exposure inequalities in London, UK. *Environmental research* 2021;198.

31 Koukouli ME, Skoulidou I, Karavias A, et al. Sudden changes in nitrogen dioxide emissions over Greece due to lockdown after the outbreak of COVID-19. *Atmospheric Chemistry and Physics* 2021;21:1759–74.

32 Lee JD, Drysdale WS, Finch DP, Wilde SE, Palmer PI. UK surface NO2 levels dropped by 42% during the COVID-19 lockdown: Impact on surface O3. *Atmospheric Chemistry and Physics* 2020;20:15743–59.

33 Lonati G, Riva F. Regional Scale Impact of the COVID-19 Lockdown on Air Quality: Gaseous Pollutants in the Po Valley, Northern Italy. *Atmosphere*  2021;12:264.

34 Lovarelli D, Conti C, Finzi A, Bacenetti J, Guarino M. Describing the trend of ammonia, particulate matter and nitrogen oxides: The role of livestock activities in northern Italy during Covid-19 quarantine. *Environmental Research* 2020;191:110048.

35 Lovrić M, Pavlović K, Vuković M, Grange SK, Haberl M, Kern R. Understanding the true effects of the COVID-19 lockdown on air pollution by means of machine learning. *Environmental Pollution* 2021;274:115900.

36 Malpede M, Percoco M. Lockdown measures and air quality: evidence from Italian provinces. *Letters in Spatial and Resource Sciences* 2021;14:101–10.

37 Marinello S, Lolli F, Gamberini R. The Impact of the COVID-19 Emergency on Local Vehicular Traffic and Its Consequences for the Environment: The Case of the City of Reggio Emilia (Italy). *Sustainability*  2020;13:118.

38 Mehlig D, Apsimon H, Staffell I. The impact of the UK’s COVID-19 lockdowns on energy demand and emissions. *Environmental Research Letters* 2021;16:054037.

39 Mesas-Carrascosa FJ, Porras FP, Triviño-Tarradas P, García-Ferrer A, Meroño-Larriva JE. Effect of Lockdown Measures on Atmospheric Nitrogen Dioxide during SARS-CoV-2 in Spain. *Remote Sensing* 2020;12:2210.

40 Munir S, Coskuner G, Jassim MS, Aina YA, Ali A, Mayfield M. Changes in Air Quality Associated with Mobility Trends and Meteorological Conditions during COVID-19 Lockdown in Northern England, UK. *Atmosphere* 2021;12:504.

41 Orak NH, Ozdemir O. The impacts of COVID-19 lockdown on PM10 and SO2 concentrations and association with human mobility across Turkey. *Environmental Research* 2021;197:111018.

42 Ozbay B, Koc Y. Impact of Covid-19 lock-down period on variations of air pollutants around an industrialized city of Turkey, Izmit. *Environmental Forensics* 2021;23:198–207.

43 Petetin H, Bowdalo D, Soret A, et al. Meteorology-normalized impact of the COVID-19 lockdown upon NO2 pollution in Spain. *Atmospheric Chemistry and Physics* 2020;20:11119–41.

44 Piccoli A, Agresti V, Balzarini A, et al. Modeling the Effect of COVID-19 Lockdown on Mobility and NO2 Concentration in the Lombardy Region. *Atmosphere*  2020;11:1319.

45 Potts DA, Marais EA, Boesch H, et al. Diagnosing air quality changes in the UK during the COVID-19 lockdown using TROPOMI and GEOS-Chem. *Environmental Research Letters* 2021;16:054031.

46 Prats RM, van Drooge BL, Fernández P, Marco E, Grimalt JO. Changes in Urban Gas-Phase Persistent Organic Pollutants During the COVID-19 Lockdown in Barcelona. *Frontiers in Environmental Science* 2021;9:109.

47 Querol X, Massagué J, Alastuey A, et al. Lessons from the COVID-19 air pollution decrease in Spain: Now what? *Science of The Total Environment* 2021;779:146380.

48 Rodrigo-Comino J, Senciales-González JM. A Regional Geography Approach to Understanding the Environmental Changes as a Consequence of the COVID-19 Lockdown in Highly Populated Spanish Cities. *Applied Sciences*  2021;11:2912.

49 Ropkins K, Tate JE. Early observations on the impact of the COVID-19 lockdown on air quality trends across the UK. *Science of The Total Environment* 2021;754:142374.

50 Rossi R, Ceccato R, Gastaldi M. Effect of Road Traffic on Air Pollution. Experimental Evidence from COVID-19 Lockdown. *Sustainability*  2020;12:8984.

51 Roșu A, Constantin DE, Voiculescu M, et al. Assessment of NO2 Pollution Level during the COVID-19 Lockdown in a Romanian City. *International Journal of Environmental Research and Public Health* 2021;18:544.

52 Ruberti M, Romano L. Correlation analysis of the variability of NO2 and PM tropospheric concentrations within the Greater Salento (Italy) before, during and after the COVID-19 lockdown | International Journal of Ecology & Development; 35(4):52-62, 2020.

53 Rugani B, Caro D. Impact of COVID-19 outbreak measures of lockdown on the Italian Carbon Footprint. *Science of The Total Environment* 2020;737:139806.

54 Sahraei MA, Kuşkapan E, Çodur MY. Impact of Covid-19 on Public Transportation Usage and Ambient Air Quality in Turkey. *Promet – Traffic &Transportation* 2021;33:179–91.

55 Salma I, Vörösmarty M, Gyöngyösi AZ, Thén W, Weidinger T. What can we learn about urban air quality with regard to the first outbreak of the COVID-19 pandemic? A case study from central Europe. *Atmospheric Chemistry and Physics* 2020;20:15725–42.

56 Sannino A, D’emilio M, Castellano P, Amoruso S, Boselli A. Analysis of Air Quality during the COVID-19 Pandemic Lockdown in Naples (Italy). *Aerosol and Air Quality Research* 2021;21:200381.

57 Sbai SE, Mejjad N, Norelyaqine A, Bentayeb F. Air quality change during the COVID-19 pandemic lockdown over the Auvergne-Rhône-Alpes region, France. *Air Quality, Atmosphere and Health* 2021;14:617–28.

58 Sifakis N, Aryblia M, Daras T, Tournaki S, Tsoutsos T. The impact of COVID-19 pandemic in Mediterranean urban air pollution and mobility. *Energy Sources, Part A: Recovery, Utilization, and Environmental Effects* 2021. doi:https://doi.org/10.1080/15567036.2021.1895373.

59 Tobías A, Carnerero C, Reche C, et al. Changes in air quality during the lockdown in Barcelona (Spain) one month into the SARS-CoV-2 epidemic. *Science of The Total Environment* 2020;726:138540.

60 Varotsos C, Christodoulakis J, Kouremadas GA, Fotaki EF. The Signature of the Coronavirus Lockdown in Air Pollution in Greece. *Water, Air, and Soil Pollution* 2021;232:1–12.

61 Velders GJM, Willers SM, Wesseling J, et al. Improvements in air quality in the Netherlands during the corona lockdown based on observations and model simulations. *Atmospheric Environment* 2021;247:118158.

62 Viatte C, Petit JE, Yamanouchi S, et al. Ammonia and PM2.5 Air Pollution in Paris during the 2020 COVID Lockdown. *Atmosphere*  2021;12:160.

63 Viteri G, Díaz de Mera Y, Rodríguez A, et al. Impact of SARS-CoV-2 lockdown and de-escalation on air-quality parameters. *Chemosphere* 2021;265:129027.

64 Vultaggio M, Varrica D, Alaimo MG. Impact on Air Quality of the COVID-19 Lockdown in the Urban Area of Palermo (Italy). *International Journal of Environmental Research and Public Health* 2020;17:7375.

65 Wyche KP, Nichols M, Parfitt H, et al. Changes in ambient air quality and atmospheric composition and reactivity in the South East of the UK as a result of the COVID-19 lockdown. *Science of The Total Environment* 2021;755:142526.

66 Al-Abadleh HA, Lysy M, Neil L, Patel P, Mohammed W, Khalaf Y. Rigorous quantification of statistical significance of the COVID-19 lockdown effect on air quality: The case from ground-based measurements in Ontario, Canada. *Journal of Hazardous Materials* 2021;413:125445.

67 Brodeur A, Cook N, Wright T. On the effects of COVID-19 safer-at-home policies on social distancing, car crashes and pollution. *Journal of Environmental Economics and Management* 2021;106:102427.

68 Chadwick E, Le K, Pei Z, et al. Technical note: Understanding the effect of COVID-19 on particle pollution using a low-cost sensor network. *Journal of Aerosol Science* 2021;155:105766.

69 Chen LWA, Chien LC, Li Y, Lin G. Nonuniform impacts of COVID-19 lockdown on air quality over the United States. *Science of The Total Environment* 2020;745:141105.

70 El-Sayed MMH, Elshorbany YF, Koehler K. On the impact of the COVID-19 pandemic on air quality in Florida. *Environmental Pollution* 2021;285:117451.

71 Elshorbany YF, Kapper HC, Ziemke JR, Parr SA. The Status of Air Quality in the United States During the COVID-19 Pandemic: A Remote Sensing Perspective. *Remote Sensing* 2021;13:369.

72 Ghosal R, Saha E. Impact of the COVID-19 induced lockdown measures on PM2.5 concentration in USA. *Atmospheric Environment (Oxford, England : 1994)* 2021;254:118388.

73 Griffin D, McLinden CA, Racine J, et al. Assessing the Impact of Corona-Virus-19 on Nitrogen Dioxide Levels over Southern Ontario, Canada. *Remote Sensing* 2020;12:4112.

74 Hudda N, Simon MC, Patton AP, Durant JL. Reductions in traffic-related black carbon and ultrafine particle number concentrations in an urban neighborhood during the COVID-19 pandemic. *Science of The Total Environment* 2020;742:140931.

75 Jia C, Fu X, Bartelli D, Smith L. Insignificant Impact of the “Stay-At-Home” Order on Ambient Air Quality in the Memphis Metropolitan Area, U.S.A. *Atmosphere* 2020;11:630.

76 Liu Q, Harris JT, Chiu LS, et al. Spatiotemporal impacts of COVID-19 on air pollution in California, USA. *Science of The Total Environment* 2021;750:141592.

77 Mashayekhi R, Pavlovic R, Racine J, et al. Isolating the impact of COVID-19 lockdown measures on urban air quality in Canada. *Air Quality, Atmosphere and Health* 2021;14:1549–70.

78 Naeger AR, Murphy K. Impact of COVID-19 Containment Measures on Air Pollution in California. *Aerosol and Air Quality Research* 2020;20:2025–34.

79 Parker HA, Hasheminassab S, Crounse JD, Roehl CM, Wennberg PO. Impacts of Traffic Reductions Associated With COVID-19 on Southern California Air Quality. *Geophysical Research Letters* 2020;47:e2020GL090164.

80 Shehzad K, Bilgili F, Koçak E, Xiaoxing L, Ahmad M. COVID-19 outbreak, lockdown, and air quality: fresh insights from New York City. *Environmental Science and Pollution Research* 2021;28:41149–61.

81 Straka W, Kondragunta S, Wei Z, Zhang H, Miller SD, Watts A. Examining the Economic and Environmental Impacts of COVID-19 Using Earth Observation Data. *Remote Sensing* 2020;13:5.

82 Tian X, An C, Chen Z, Tian Z. Assessing the impact of COVID-19 pandemic on urban transportation and air quality in Canada. *Science of The Total Environment* 2021;765:144270.

83 Wong MS, Zhu R, Yin Tung Kwok C, et al. Association between NO2 concentrations and spatial configuration: a study of the impacts of COVID-19 lockdowns in 54 US cities. *Environmental Research Letters* 2021;16:054064.

84 Acharya P, Barik G, Gayen BK, et al. Revisiting the levels of Aerosol Optical Depth in south-southeast Asia, Europe and USA amid the COVID-19 pandemic using satellite observations. *Environmental research* 2021;193.

85 Bauwens M, Compernolle S, Stavrakou T, et al. Impact of Coronavirus Outbreak on NO2 Pollution Assessed Using TROPOMI and OMI Observations. *Geophysical Research Letters* 2020;47:e2020GL087978.

86 Chossière GP, Xu H, Dixit Y, et al. Air pollution impacts of COVID-19–related containment measures. *Science Advances* 2021;7.

87 Collivignarelli MC, De Rose C, Abbà A, et al. Analysis of lockdown for CoViD-19 impact on NO 2 in London, Milan and Paris: What lesson can be learnt? *Process safety and environmental protection : transactions of the Institution of Chemical Engineers, Part B* 2021;146:952–60.

88 Connerton P, de Assunção JV, de Miranda RM, Slovic AD, Pérez-Martínez PJ, Ribeiro H. Air Quality during COVID-19 in Four Megacities: Lessons and Challenges for Public Health. *International Journal of Environmental Research and Public Health* 2020;17:5067.

89 Fu F, Purvis-Roberts KL, Williams B. Impact of the COVID-19 Pandemic Lockdown on Air Pollution in 20 Major Cities around the World. *Atmosphere* 2020;11:1189.

90 Goldberg DL, Anenberg SC, Griffin D, McLinden CA, Lu Z, Streets DG. Disentangling the Impact of the COVID-19 Lockdowns on Urban NO2 From Natural Variability. *Geophysical Research Letters* 2020;47:e2020GL089269.

91 Gope S, Dawn S, Das SS. Effect of COVID-19 pandemic on air quality: a study based on Air Quality Index. *Environmental science and pollution research international* 2021;28:35564–83.

92 He C, Hong S, Zhang L, et al. Global, continental, and national variation in PM2.5, O3, and NO2 concentrations during the early 2020 COVID-19 lockdown. *Atmospheric Pollution Research* 2021;12:136–45.

93 Kumari P, Toshniwal D. Impact of lockdown on air quality over major cities across the globe during COVID-19 pandemic. *Urban Climate* 2020;34:100719.

94 Liu Q, Malarvizhi AS, Liu W, et al. Spatiotemporal changes in global nitrogen dioxide emission due to COVID-19 mitigation policies. *Science of The Total Environment* 2021;776:146027.

95 Liu Z, Ciais P, Deng Z, et al. Near-real-time monitoring of global CO2 emissions reveals the effects of the COVID-19 pandemic. *Nature Communications* 2020;11:1–12.

96 Rodríguez-Urrego D, Rodríguez-Urrego L. Air quality during the COVID-19: PM2.5 analysis in the 50 most polluted capital cities in the world. Environmental Pollution. 2020;266:115042.

97 Sahraei MA, Kuşkapan E, Çodur MY. Public transit usage and air quality index during the COVID-19 lockdown. *Journal of environmental management* 2021;286.

98 Sannigrahi S, Kumar P, Molter A, et al. Examining the status of improved air quality in world cities due to COVID-19 led temporary reduction in anthropogenic emissions. *Environmental Research* 2021;196:110927.

99 Shakoor A, Chen X, Farooq TH, et al. Fluctuations in environmental pollutants and air quality during the lockdown in the USA and China: two sides of COVID-19 pandemic. *Air quality, atmosphere, & health* 2020;13:1335–42.

100 Shi Z, Song C, Liu B, et al. Abrupt but smaller than expected changes in surface air quality attributable to COVID-19 lockdowns. *Science advances* 2021;7.

101 Sicard P, De Marco A, Agathokleous E, et al. Amplified ozone pollution in cities during the COVID-19 lockdown. *Science of The Total Environment* 2020;735:139542.

102 Singh RK, Drews M, De la Sen M, et al. Highlighting the compound risk of COVID-19 and environmental pollutants using geospatial technology. *Scientific Reports* 2021;11:1–12.

103 Skirienė AF, Stasiškienė Ž. COVID-19 and Air Pollution: Measuring Pandemic Impact to Air Quality in Five European Countries. *Atmosphere* 2021;12:290.

104 Solberg S, Walker SE, Schneider P, Guerreiro C. Quantifying the impact of the covid-19 lockdown measures on nitrogen dioxide levels throughout europe. *Atmosphere* 2021;12:1–20.

105 Steinbrecht W, Kubistin D, Plass-Dülmer C, et al. COVID-19 Crisis Reduces Free Tropospheric Ozone Across the Northern Hemisphere. *Geophysical research letters* 2021;48.

106 Venter ZS, Aunan K, Chowdhury S, Lelieveld J. COVID-19 lockdowns cause global air pollution declines. *Proceedings of the National Academy of Sciences of the United States of America* 2020;117:18984–90.

107 Vîrghileanu M, Săvulescu I, Mihai BA, Nistor C, Dobre R. Nitrogen Dioxide (NO2) Pollution Monitoring with Sentinel-5P Satellite Imagery over Europe during the Coronavirus Pandemic Outbreak. *Remote Sensing* 2020;12:3575.

108 Wang H, Tan J, Li X. Global NO2 Dynamics During the COVID-19 Pandemic: A Comparison Between Two Waves of the Coronavirus. *IEEE Journal Of Selected Topics In Applied Earth Observations And Remote Sensing* 2021;14:4310–20.

109 Wang Q, Li S. Nonlinear impact of COVID-19 on pollutions – Evidence from Wuhan, New York, Milan, Madrid, Bandra, London, Tokyo and Mexico City. *Sustainable Cities and Society* 2021;65:102629.
