# Supplementary figures and images for: The impact of COVID-19 lockdown on air pollution in Europe and North America: a systematic review
Source: Eur J Public Health. 2022 Sep 8;32(6):962–8. doi: 10.1093/eurpub/ckac118 (PMC9494388; doi:10.1093/eurpub/ckac118)

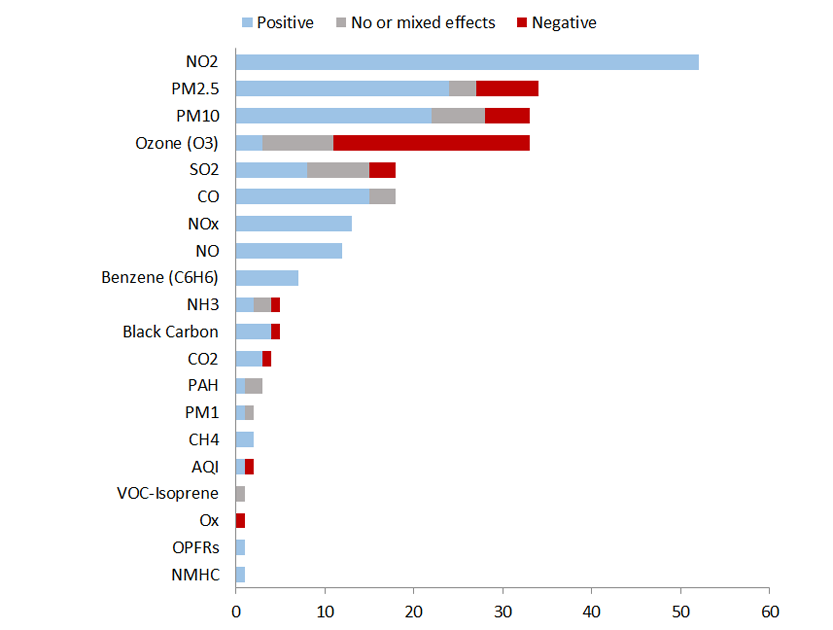

Supplement: ckac118_Supplementary_Data [file ckac118_supplementary_data.zip › ejph-2022-05-om-0244-File010.tif]

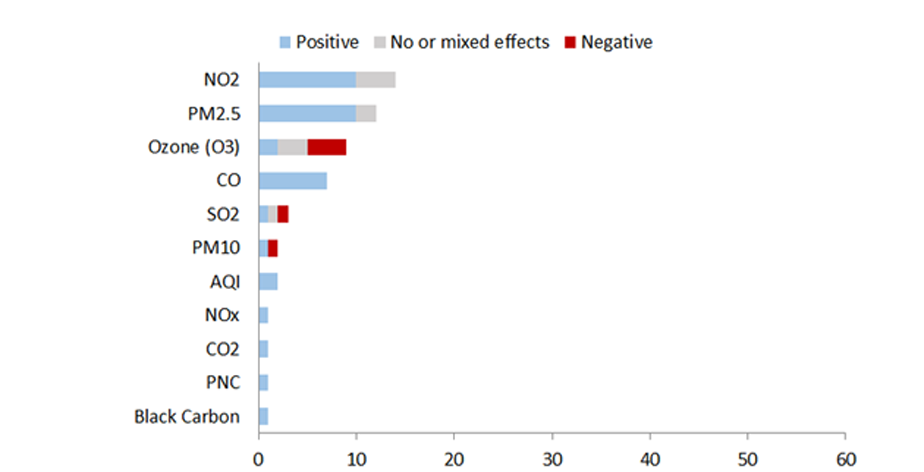

Supplement: ckac118_Supplementary_Data [file ckac118_supplementary_data.zip › ejph-2022-05-om-0244-File011.tif]

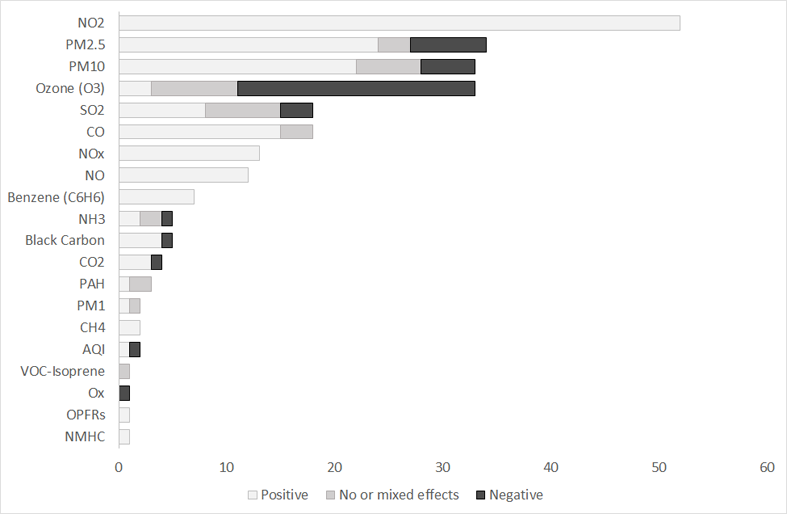

Supplement: ckac118_Supplementary_Data [file ckac118_supplementary_data.zip › Figure_Europe[AU].tif]

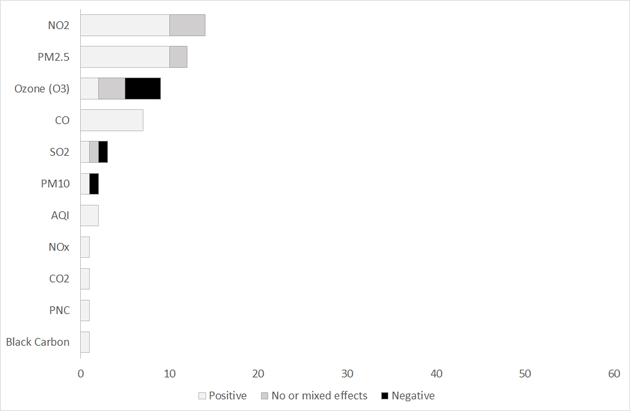

Supplement: ckac118_Supplementary_Data [file ckac118_supplementary_data.zip › Figure_NorthAmerica[AU].tif]
